# New Blood Coagulation Factor XIIa Inhibitors: Molecular Modeling, Synthesis, and Experimental Confirmation

**DOI:** 10.3390/molecules27041234

**Published:** 2022-02-12

**Authors:** Anna Tashchilova, Nadezhda Podoplelova, Alexey Sulimov, Danil Kutov, Ivan Ilin, Mikhail Panteleev, Khidmet Shikhaliev, Svetlana Medvedeva, Nadezhda Novichikhina, Andrey Potapov, Vladimir Sulimov

**Affiliations:** 1 Dimonta, Ltd., 117186 Moscow, Russia; at@dimonta.com (A.T.); sulimovv@mail.ru (A.S.); ivan.ilyin@srcc.msu.ru (I.I.); 2 Research Computing Center, Lomonosov Moscow State University, 119992 Moscow, Russia; 3 Russian Children’s Clinical Hospital of the Pirogov Russian National Research Medical University of the Ministry of Healthcare of the Russian Federation, 119571 Moscow, Russia; podoplelovan@yandex.ru (N.P.); mapanteleev@yandex.ru (M.P.); 4 Center for Theoretical Problems of Physicochemical Pharmakology, 119991 Moscow, Russia; 5 Department of Organic Chemistry, Faculty of Chemistry, Voronezh State University, 1 Universitetskaya sq., 394018 Voronezh, Russia; chocd261@chem.vsu.ru (K.S.); smmedvedeva@rambler.ru (S.M.); podaneva_nadya@mail.ru (N.N.); pistones@mail.ru (A.P.)

**Keywords:** anticoagulants, coagulation factor XIIa, docking, quantum chemistry, protein-ligand binding

## Abstract

In the modern world, complications caused by disorders in the blood coagulation system are found in almost all areas of medicine. Thus, the development of new, more advanced drugs that can prevent pathological conditions without disrupting normal hemostasis is an urgent task. The blood coagulation factor XIIa is one of the most promising therapeutic targets for the development of anticoagulants based on its inhibitors. The initial stage of drug development is directly related to computational methods of searching for a lead compound. In this study, docking followed by quantum chemical calculations was used to search for noncovalent low-molecular-weight factor XIIa inhibitors in a focused library of druglike compounds. As a result of the study, four low-molecular-weight compounds were experimentally confirmed as factor XIIa inhibitors. Selectivity testing revealed that two of the identified factor XIIa inhibitors were selective over the coagulation factors Xa and XIa.

## 1. Introduction

Disorders in the blood coagulation system remain the most common cause of death and disability in the world. Thrombotic and hemorrhagic complications occur in different fields of medicine, including oncology, hematology, and immunology. Thus, the development of new drugs that can prevent such pathological conditions without disrupting normal hemostasis is an urgent need today.

Compounds targeting nearly every enzyme in the blood coagulation cascade are being developed to identify new anticoagulants that are at least as effective as existing drugs and without the risk of bleeding complications. Among them, factor XIIa or factor XIa are promising targets for creating drugs based on their inhibitors, since a deficiency of these factors protects against thrombosis without causing spontaneous bleeding [1,2,3,4].

**Figure 1:**
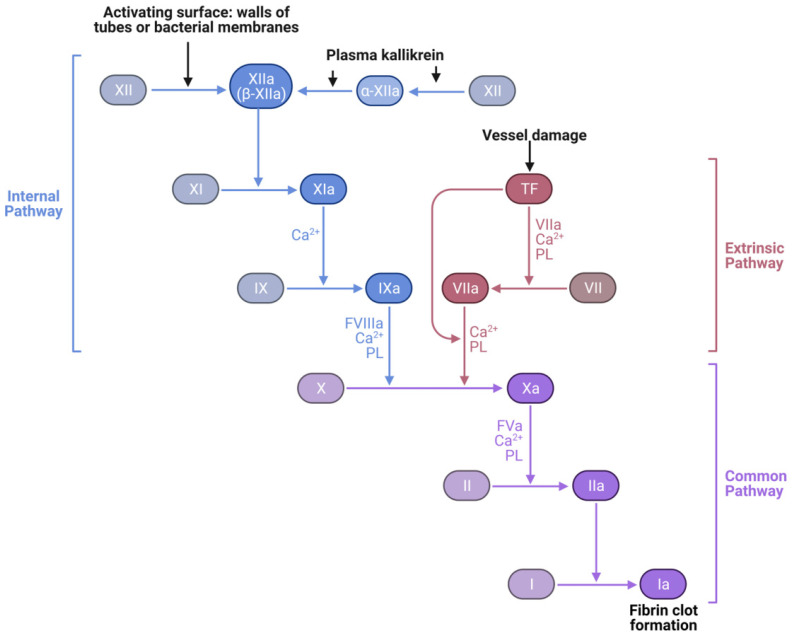
Reactions of the plasma blood coagulation system. Roman numerals—coagulation factors; TF—tissue factor; PL—phospholipids; Ca^2+^—calcium ions. The reactions of converting coagulation factors into active forms are shown by horizontal arrows; vertical arrows indicate which reaction is catalyzed by each factor. Adapted from “Coagulation Cascade” by BioRender.com (2021). Retrieved from https://app.biorender.com/biorender-templates (accessed on 9 January 2022).

Plasma coagulation is a cascade of biochemical reactions that sequentially activates serine proteases, coagulation factors, and leads to fibrin polymerization and the formation of a clot. The reactions of the plasma system are conventionally divided into external (tissue factor pathway) and internal (contact) pathways (see Figure 1).

In the contact or internal coagulation pathway, factor XII is activated upon contact with a surface (negatively charged—such surfaces can be the walls of test tubes, catheters, etc.) or with bacterial membranes during infections, and activated factor XIIa activates factor XI. Factor XIa activates factor IX in the presence of calcium ions, which in turn activates factor X. The activation of the latter also requires calcium ions as well as a phospholipids surface. In the complex with factor VIIa and calcium ions, factor Xa initiates the reaction of the conversion of prothrombin (factor II) to thrombin (factor IIa), which, in turn, converts soluble fibrinogen (factor I) into insoluble fibrin (factor Ia), which, upon polymerization, leads to blood coagulation. Thrombin also activates factor XI, which realizes the additional activation of factor IX.

The extrinsic pathway is also called the tissue factor pathway (TF), since it begins with the contact of blood plasma with a protein TF. This path is called external because there is no TF in the vascular endothelium and in the blood cells. The vessel must be damaged for the blood to come into contact with tissue factor. In the presence of a phospholipids surface and calcium ions, TF activates factor VIIa. TF and factor VIIa constitute a tenase complex which catalyzes the activation of factor X and, because of further sequential activation of coagulation factors, also leads to clot formation. Thus, the position of coagulation factor XIIa in the cascade of biochemical reactions will make it possible to create, on the basis of its inhibitors, a new class of anticoagulants capable of blocking the internal pathway of coagulation activation, while not disrupting the chain of reactions of plasma hemostasis activated by the external pathway [1,2,3,4].

In this study, coagulation factor XIIa was chosen as a target for the development of low-molecular-weight inhibitors. This protein was discovered a long time ago, but its in vivo function and three-dimensional structure began to be discovered relatively recently. With factor XII deficiency, laboratory animals are either completely protected from the development of induced thrombosis or suffer significantly less from its consequences. This discovery was tested in a dozen works on a variety of experimental models: in mice, rats, rabbits, and even primates, in which factor XII deficiency was caused either by gene knockout, the suppression of its expression in hepatocytes using antisense oligonucleotides, or the use of direct inhibitors of factor XIIa and antibodies that suppress the activity of factor XIIa [5,6,7,8]. Initially, the crystal structure of the catalytic domain of recombinant human factor XII (amino acid residues 354–596) was identified [9]. However, the revealed structure of the zymogen of the catalytic domain of factor XII did not provide the necessary information about the active site of the protein, which makes it impossible to develop drugs using molecular modeling.

The inactive form of coagulation factor XII is a polypeptide chain of 596 amino acid residues. Upon activation of the contact coagulation system, inactive factor XII is cleaved at the Arg353-Val354 peptide bond under the action of plasma kallikrein to form α-FXIIa, consisting of heavy and light chains linked by a Cys340-Cys467 disulfide bond. Further cleavage of the α-FXIIa heavy chain at the C-termini of Arg334 and Arg343 by plasma kallikrein leads to the formation of β-FXIIa [10]. β-FXIIa is a trypsin-like serine protease consisting of two polypeptide chains: a heavy chain and a catalytic domain, covalently linked by a disulfide bond.

In a recent study [11], the crystal structures of factor β-XIIa were determined in complexes with two inhibitors: a noncovalent benzamidine inhibitor and a covalent synthetic inhibitor. These structures can be the basis for creating models of coagulation factor XIIa, which will allow the initiation of the search for low-molecular-weight inhibitors of this target by means of structure-oriented virtual screening based on docking.

**Figure 2:**
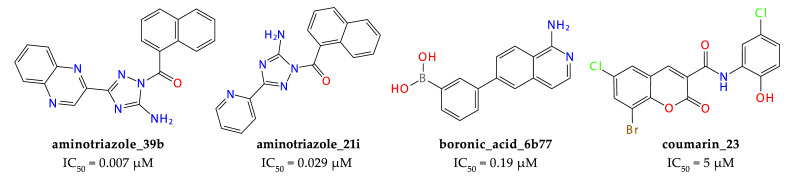
Factor XIIa inhibitors reported in literature.

Currently, there are various inhibitors of the coagulation factor XIIa tested through in vitro and in vivo experiments, including monoclonal antibodies [8,12], natural peptide or protein inhibitors [5], a synthetic peptide macrocycle inhibitor [13], RNA aptamer [14], small interfering RNA [15], and antisense oligonucleotide [16]. The review [17] lists various identified inhibitors of coagulation factor XIIa, including low-molecular-weight inhibitors. Weak but selective inhibitors of factor XIIa based on 3,6-disubstituted coumarins have been identified using scaffold-based discovery [18,19]. In a study using virtual high-throughput screening [20], 17 factor XIIa inhibitors with IC_50_ values below 50 μM were identified. Figure 2 shows the structures of the reported coagulation factor XIIa inhibitors and their in vitro inhibitory activity values: inhibitors based on aminotriazole selective over thrombin, trypsin, and FXa [21,22]; inhibitors based on coumarin selective over thrombin, plasma kallikrein, TF/FXVIIa, and FXa [18]; boronic-acid-based inhibitor [11]. Nevertheless, the development of inhibitors of coagulation factor XIIa is at an early stage and the search for a lead compound to create a drug based on it with a low risk of bleeding is an urgent task today [1]. So far, none of the existing factor XIIa inhibitors has entered the stage of clinical trials in humans. 

In this study, using the methods of structure-oriented virtual screening with the SOL docking program [23,24] and the subsequent calculation of the enthalpy of protein–ligand binding by the PM7 quantum chemical semiempirical method [25] with the COSMO implicit solvent model [26] using the MOPAC program [27], thirteen compounds were identified as potential inhibitors of blood coagulation factor XIIa. Experimental in vitro testing confirmed micromolar activity for four of the thirteen selected compounds. The IC_50_ for the best inhibitor, **225006**, was 7.9 µM. The selectivity over coagulation factors Xa and XIa was measured for the four identified inhibitors of factor XIIa. It was shown that **225006** is a selective, low-micromolar inhibitor of blood coagulation factor XIIa over these two off-targets.

## 2. Results and Discussion

### 2.1. Factor XIIa Structure Preparation

**Figure 3:**
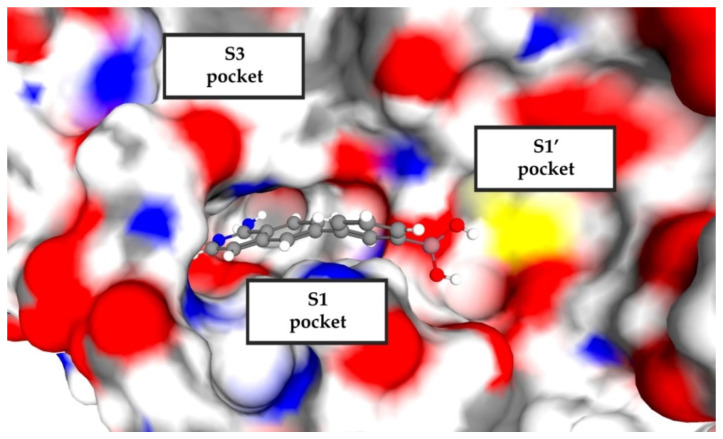
The active site of coagulation factor XIIa (PDB ID: 6B77) with the crystallized native ligand; the protein active site with pockets S1, S1’, and S3 is represented by the solvent accessible surface, and different areas of the surface are colored in accordance with the types of protein atoms located nearby: red—oxygen; gray—carbon; blue—nitrogen; white—hydrogen; and yellow—sulfur atoms.

To create a model of coagulation factor XIIa, we used a crystal structure from the protein bank PDB (Protein Data Bank) [28]. At the time of this study, five structures of factor XIIa had been found, obtained by X-ray structural analysis: 6QF7, 6GT6, 6B77, 6B74, 6L63. To build a model, a high-quality protein structure is required: resolution < 2.5 Å, no missing atoms or amino acid residues. The structure of the protein in a complex with a noncovalently bound small molecule (a ligand) is also preferred. Among the structures found in the Protein Data Bank, only one (6B74) met the above criteria. The 6B74 complex was obtained by X-ray diffraction analysis with a resolution of 2.3 Å, had no missing atoms or amino acid residues, and was crystallized in a complex with benzamidine [7]. The 6B74 complex contains two chains: A and B, corresponding to a heavy chain and a catalytic domain of factor XIIa. The catalytic triad consists of three amino acid residues: His57, Asp102, and Ser195. The active site of the protein is represented by binding pockets—S1–S4, S1’–S4’; the deepest are shown in Figure 3.

To validate the docking protocol and the protein model, docking of the native ligand was carried out. This refers to the positioning of the original ligand inhibitor in the protein with which it is cocrystallized. This aims to check the suitability of the selected docking software for the modeling of a binding process to the target protein and validate whether the prepared protein model is good enough to reproduce an experimental inhibitor pose.

The docking of the native ligand 6B74 was successful, since the RMSD from the native ligand was <2 Å and the clustering analysis of the docking solutions showed a high population of the first cluster (49 poses), which indicates a high probability of finding the global minimum energy of the system, since with 50 independent launches of the global optimization algorithm, 49 cases resulted in the ligand pose with the best energy, and all these poses differ from each other by only RMSD < 1 Å. The scoring function of the SOL program was −5.19 kcal/mol. The prepared model of factor XIIa was chosen for further search for inhibitors.

### 2.2. Virtual Screening

At the first stage, virtual screening based on docking was carried out by the SOL program and the subsequent calculation of the enthalpy of protein–ligand binding by the PM7+COSMO method for the focused library of compounds selected from the database of druglike substances of the Department of Organic Chemistry of the Voronezh State University (VSU). This library consisted of 59 compounds, which were all previously tested against coagulation factors Xa and XIa. Among them, there were a few confirmed inhibitors of these proteins with micromolar activity. The presence of inhibition data for these off-targets allowed us to use selectivity data after the screening phase and better understand the quality of the identified hit series. Screening and testing the selected compounds from the focused library also helped clarify the threshold values of the scoring function of the SOL program, the binding enthalpy and, accordingly, adjust the screening process for further iterations.Virtual screening with the SOL program was carried out for 326 three-dimensional molecules. The original base of 59 ligands was expanded taking into account a few protomers, isomers, and conformers of these molecules. At the first stage, the selection of compounds was carried out based on the SOL scoring function. All compounds that had a scoring SOL > −5.00 kcal/mol were discarded. Note that the SOL scoring value for the docking of the original ligand (PDB ID 6B74) was −5.19 kcal/mol. Thus, the selection criterion covered a wider range of scoring values.

Further, for the 39 compounds selected on the basis of the scoring function of the SOL program, the enthalpy of binding, ΔH_bind_, was calculated by the MOPAC program [27]; visual analysis of the docking pose in the active site of factor XIIa and clustering analysis were also used as selection criteria.

As a result, thirteen compounds were selected for experimental testing. Since the selection of compounds was carried out for the first experimental verification, the main task was the experimental testing of chemically diverse structures with acceptable values of the SOL scoring function and the enthalpy of protein–ligand binding. All compounds selected for the experiment had a population of the first cluster > 12, and the occupation of the binding pockets S1, S1’, and S3 of the active site of factor XIIa.

### 2.3. Results of Experimental Testing

**Figure 4:**
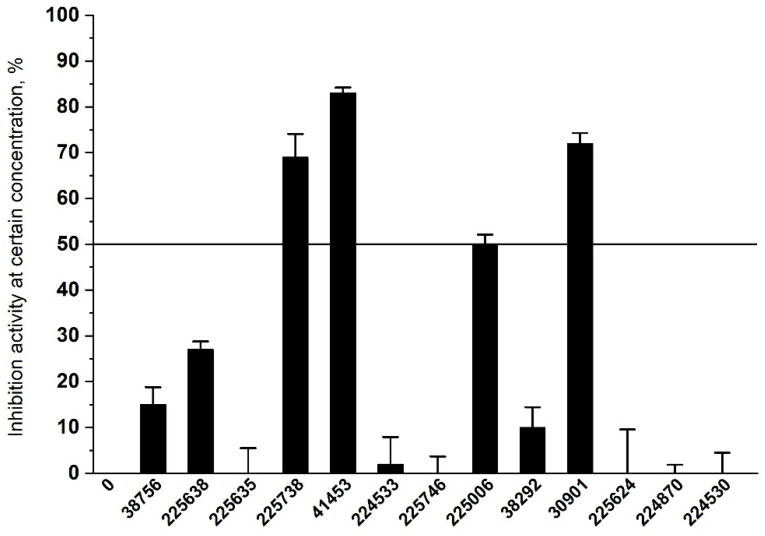
Results of the experimental testing of the activity of coagulation factor XIIa for 13 inhibitors selected from the focused library. Experiments were performed using an inhibitor concentration of 30 μM. The root-mean-square error from three independent measurements (±SEM) for four active compounds was 0.012–0.051.

According to experimental testing, we observed the activity of four compounds: **30901**, **41453**, **225006**, and **225738**, for which the inhibition activity values (inhibition activity is the % of inhibition at a certain concentration) were greater than 50%. Table 1 and Figure 4 depict the results of calculations and experimental in vitro testing of the thirteen selected inhibitors.

All thirteen compounds presented in Table 1 and in Figure 4 were selected from a focused library using two criteria: sufficiently negative SOL scores and protein–ligand binding enthalpies. Among the four experimentally confirmed inhibitors, the first three ligands in Table 1 satisfy these criteria, but the fourth inhibitor, **225006**, had noticeably low absolute binding enthalpy (−15.5 kcal/mol). This anomaly can be attributed to the fact that this inhibitor may very likely be a covalent inhibitor, since it contains a reactive group—a Michael acceptor as part of the coumarin fragment. It is interesting that this fragment is also found in factor XIIa inhibitors reported in the literature (see Figure 2). Among the other nine experimentally inactive compounds, there were several false positives with sufficiently negative SOL scores and binding enthalpies. This indicates the need for further improvement of the methodology for the selection of factor XIIa inhibitors, especially in terms of calculating the enthalpy of protein–ligand binding using the PM7 quantum chemical method and the COSMO solvent model. In terms of predicted physicochemical properties related to primary ADME profile, we can note that only one compound, **41453**, fails logP criteria for Lipinski rule of five [29]. All predicted topological polar surface areas are less than 140 Å^2^ and all compounds have fewer 10 rotatable bonds (not shown) that means all they have a high probability of good oral bioavailability in the rat according to the study [30]. Five compounds (**30901**, **225006**, **225638**, **38756**, **225635**) meet the Pfizer 3/75 rule (the rule states that a compound is supposed to have a 6-fold reduced preclinical toxicity when logP < 3 and TPSA > 75 Å) [31].

**Figure 5:**
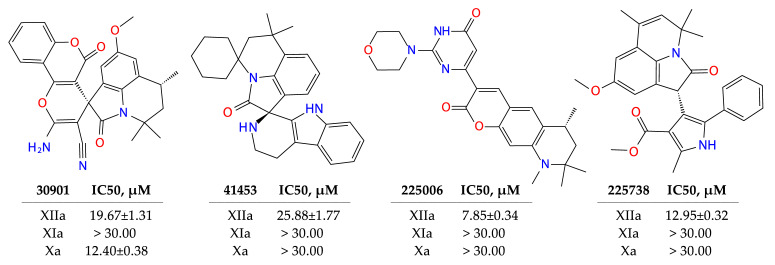
Structures of four identified inhibitors of the coagulation factor XIIa. IC_50_ values are shown for three coagulation factors: Xa, XIa, and XIIa. For compounds which did not show any activity against off-target at 30 µM, IC_50_ was not measured. So, they are marked with ‘>30’ in a column with activity values.

Figure 5 contains the structures of four inhibitors of factor XIIa and IC_50_ values for three coagulation factors, Xa, XIa, and XIIa. According to Figure 5, compounds **225006** and **225738** selectively inhibit factor XIIa over coagulation factors Xa and XIa. The activity of **41453** against factor XIIa is on the border of the concentration range used for primary testing against factor Xa and XIa. So, its selectivity is questionable. It is also noteworthy that the three inhibitors depicted (**30901**, **41453, 225738**) have a similar scaffold. Inhibition curves can be found in Supplementary Materials (Figures S18–S22).

**Figure 6:**
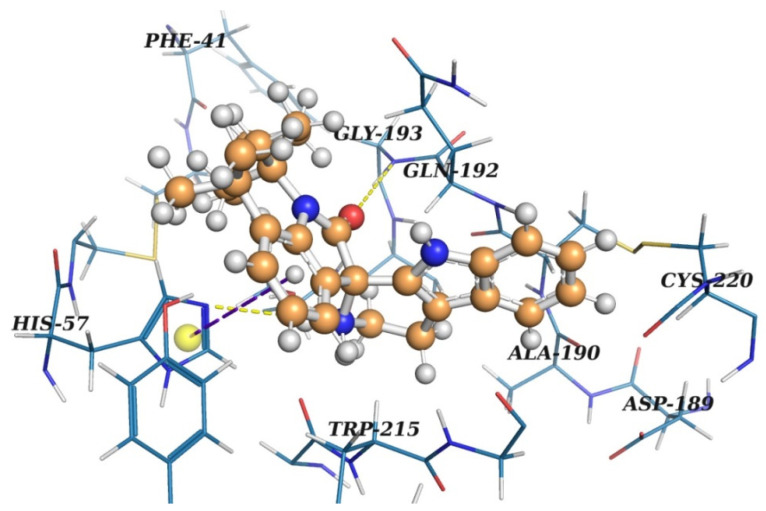
Binding mode of compound **41453** with factor XIIa predicted by docking. A pose after local optimization by PM7 is used. Interactions predicted by PLIP. H-bonds are shown as dashed yellow lines. A dashed violet line indicates pi-stacking. The ligand is presented in a ‘ball-and-stick’ model and colored in orange. Protein residues are shown in a ‘line’ model and colored in marine.

Compound **41453** showed an IC_50_ around 26 µM. Its docking pose after local optimization in PM7 is depicted in Figure 6 (it was prepared in PyMOL [33]). As can be seen from Figure 6, compound **41453** fits the active site of factor XIIa well, with indole moiety buried in the S1 pocket and a cyclohexane ring placed toward the S1′ pocket. According to visual inspection and PLIP [34] prediction, it forms three specific interactions with factor XIIa: H-bond with Gly-193NH and H-bond and pi-stacking with an imidazole ring of His-57. To estimate the strength of these H-bonds quantitatively, we performed PM7 quantum chemical calculations using the MOPAC program, printing all the H-bonds detected for the complex of factor XIIa with compound **41453**. The H-bond between a carbonyl oxygen of **41453** and Gly-193NH turned out to be quite strong (−1.81 kcal/mol) despite the nonideal geometry observed visually. The energy of the second H-bond involving a charged nitrogen of **41453** and His-57 was less than −1.0 kcal/mol. So, energetically, this H-bond, while predicted by PLIP, is unfavorable and does not exist. This seems to be due to the long distance between hydrogen and an acceptor (2.85 Å) and the bad orientation of the lone pair of an acceptor—it is not placed properly toward a hydrogen atom. Thus, of the two H-bonds predicted with the qualitative approach, only one had enough energy to contribute to the stabilization of the predicted protein–ligand complex.

**Figure 7:**
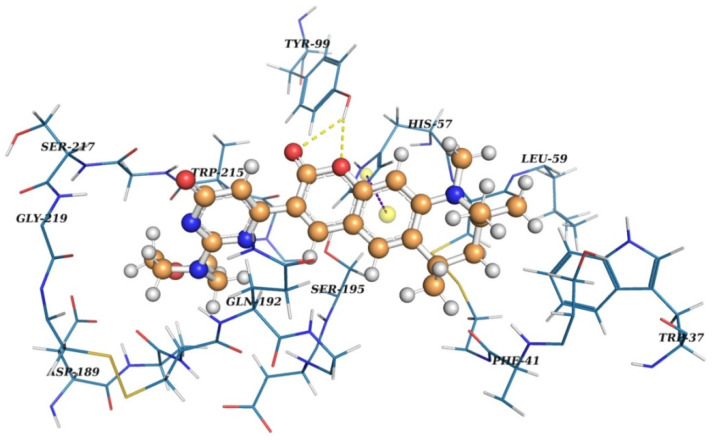
Binding mode of compound **225006** with factor XIIa predicted by docking. A pose after local optimization in PM7 is used. Interactions are predicted by PLIP. H-bonds are shown as dashed yellow lines. A dashed blue line indicates pi-stacking. The ligand is presented in a ‘ball-and-stick’ model and colored in orange. Protein residues are shown in a ‘line’ model and colored in marine.

Compound **225006** showed the most potent action on factor XIIa. It is a potential covalent inhibitor since it contains an electrophilic reactive group—a Michael acceptor as part of the coumarin fragment. The proximity between a Michael acceptor and catalytic Ser-195 is predicted in docking (see Figure 7). In its docked pose, compound **225006** forms an H-bond with the hydroxyl group of Tyr-99 and pi-stacking with His-57. The presence of the H-bond is also confirmed by quantum chemical calculations—its energy is estimated at −1.23 kcal/mol, according to the PM7 method.

Despite its high potential of forming a covalent bond with the protein, compound **225006** possesses relatively weak activity. This can be attributed to the bad contacts observed after modeling between the morpholine ring of compound **225006** buried in a cavity with Asp-189 and the protein backbone, making binding less favorable. It is interesting that the SOL score was not sensitive to these clashes and was quite negative for the predicted pose of compound **225006** (score = −6.55 kcal/mol), whereas the binding enthalpy calculated with the PM7 method was evidently bad (ΔH_bind_ = −15.5 kcal/mol). This might be because, as many docking programs do, SOL uses the broadening of VdW potentials to implicitly account for the limited mobility of protein atoms and only penalize conformations with very close collisions between protein and ligand atoms.

## 3. Materials and Methods

### 3.1. Preparation of Protein and Ligands

Protein preparation for docking with the SOL program included the addition of hydrogen atoms to the protein structure at pH = 7.4 using the Aplite program [23,35]. Each atom was assigned to a certain type in accordance with the MMFF94 force field [36]. Then, using the Aplite program, the coordinates of the protein center were determined as an input file for the SOLGRID [23,24] module. We used the geometric center of the native ligand cocrystallized with the corresponding protein to determine the center of docking cube. With the help of the SOLGRID module, a grid of potentials, described below, was calculated for subsequent docking. It was also verified that the constructed cube captured the entire active site of the target protein.

The preparation of libraries of chemical compounds, as well as individual ligands for docking with the SOL program, involved protonation at pH = 7.4 using the module ChemAxon Protonation [32]. The 2D structures of the ligands were converted to 3D structures using the ChemAxon Generate3D module. Each low-energy conformer of nonaromatic rings and macrocycles present in one molecule was considered as a separate ligand, since the SOL program samples conformational space of a ligand, varying only its internal rotational degrees of freedom (torsions).

### 3.2. Ligand Docking

In this study, docking was carried out by the SOL program [23,24]. SOL uses a precalculated grid of potentials that describes the interactions of ligand atoms with a rigid protein. The potentials include the Coulomb and Van der Waals interactions described by the MMFF94 force field, and the desolvation potentials which are calculated using a simplified form of the generalized Born solvent model. These potentials are calculated in each node of the grid in the docking cube with an edge length of 22 Å and a distance between adjacent nodes equal to 0.22 Å. This size of the docking cube is large enough to cover the entire active site of the target protein with a noticeable excess. The SOL program was adapted for virtual screening on the Lomonosov-2 supercomputer of Lomonosov Moscow State University. Depending on the size and number of ligand torsions, the docking of one ligand on one computational core takes between one and several hours.

For global energy optimization, a genetic algorithm was used with the following main parameters [23,24]: population size—30,000; number of generations—1,000. For each ligand, 50 independent runs of the genetic algorithm were performed. The fifty solutions found were grouped into clusters using a criterion RMSD < 1 Å, where RMSD is the root-mean-square deviation between two different ligand poses calculated over all ligand atoms. An indicator of reliable finding of the solution of the global optimization problem is a relatively high population of the cluster containing a ligand pose with the lowest value of the target energy function. We considered docking successful when the population of the cluster with the lowest ligand energies was not less than 10. The target function in solving the global optimization problem was the sum of the energy of protein–ligand interaction and the ligand internal strain energy. The SOL program has been successfully used to develop inhibitors of thrombin [37], urokinase (uPA) [38,39], and coagulation factors Xa and XIa [40,41,42].

### 3.3. Protein–Ligand Binding Enthalpy

The enthalpy of protein–ligand binding was calculated by the PM7 semiempirical quantum chemical method [25] with the COSMO implicit solvent model [26], implemented in the MOPAC program [27]. The binding enthalpy was calculated according to the equation:

(1) $$\Delta H_{\mathrm{bind}}=\Delta H_{\mathrm{protein}-\mathrm{ligand}}-\left(\Delta H_{\mathrm{protein}}+\Delta H_{\mathrm{ligand}}\right),$$

$\Delta H_{\mathrm{protein}-\mathrm{ligand}}$ is the enthalpy of formation of the protein–ligand complex; $\Delta H_{\mathrm{ligand}}$ is the enthalpy of formation of the unbound ligand; $\Delta H_{\mathrm{protein}}$ is the enthalpy of formation of unbound protein. In this equation, $\Delta H_{\mathrm{protein}}$ is calculated for the same conformation of the protein that was used for docking, $\Delta H_{\mathrm{ligand}}$ is calculated for the unbound ligand conformation with the lowest PM7+COSMO energy, and $\Delta H_{\mathrm{protein}-\mathrm{ligand}}$ is calculated as follows. The local optimization of the energy of the protein–ligand complex using the PM7 method was carried out from the initial ligand pose in the active site of the protein that was found during docking. In the course of this energy optimization, positions of all ligand atoms were varied. In the identified local minimum, the energy of the complex was recalculated using PM7 and the COSMO solvent model.

### 3.4. Synthesis

**Figure 8:**
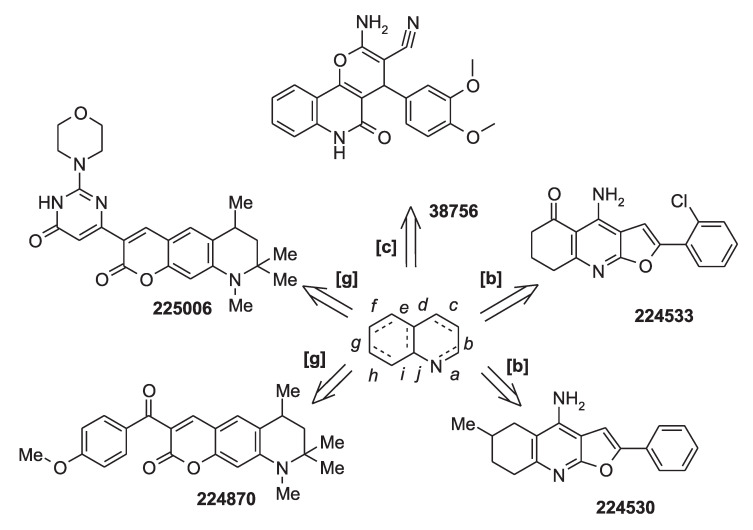
Structures of tricyclic hydroquinolines annelated at the [c], [g], and [b] bonds.

**Figure 9:**
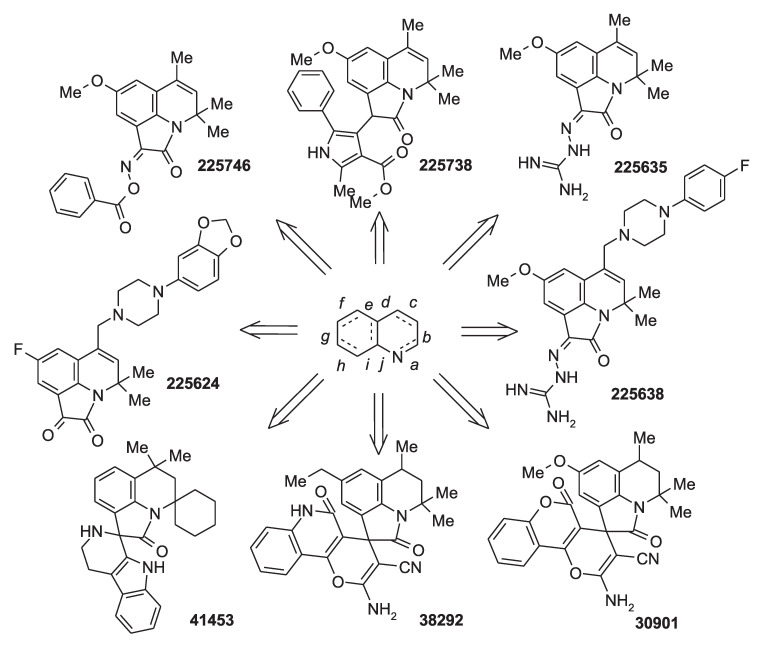
Structures of tricyclic hydroquinolines annelated at the [ij] bonds.

The strategy of the molecular design of tricyclic hydroquinoline derivatives consisted in the fact that a fragment of a partially hydrogenated quinoline cycle (with a hydrogenated heterocyclic or benzene ring) was annelated to five- and six-membered heterocycles at various bonds: [c], [g], [b], and [ij]. Structural diversification was achieved by introducing aryl substituents, as well as by linear bonding or forms a spiro system with some oxy- or aza-heterocycles. The structures of the studied tricyclic hydroquinolines are shown in Figure 8 (bonds [c], [g], and [b]) and Figure 9 ([ij] bonds).

**Scheme 1:**
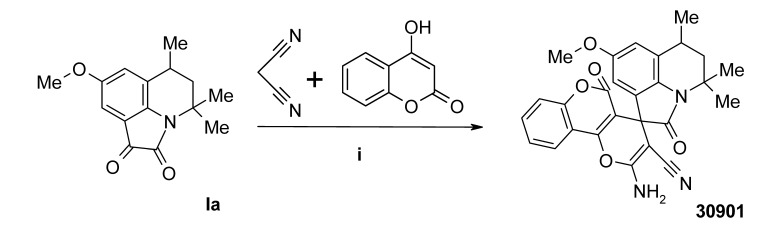
Synthetic route to 2-amino-8’-methoxy-4’,4’,6’-trimethyl-2’,5-dioxo-5’,6’-dihydro-2’*H*,4’*H*,5*H*-spiro[pyrano[3,2-*c*]chromene-4,1’-pyrrolo[3,2,1-*ij*]quinoline]-3-carbonitrile (**30901**). (i) EtOH, catalytic amount of *N*-methylpiperazine, reflux, 15 min.

Synthesis techniques and characteristics of compounds **41453** (6’-6’-dimethyl-2,3,4,5’,6’,9-hexahydrodispiro[*ß*-carboline-1,1’-pyrrolo[3,2,1-*ij*]quinoline-4’,1’’-cyclohexane]-2’-one) and **225006** (6,8,8,9-tetramethyl-3-[2-(morpholin-4-yl)-6-oxo-1,6-dihydropyrimidin-4-yl]-6,7,8,9-tetrahydro-2*H*-pyrano-[3,2-*g*]quinolin-2-one) were previously described in our works [43] and [44], respectively. Undescribed compound **30901** (2-amino-8’-methoxy-4’,4’,6’-trimethyl-2’,5-dioxo-5’,6’-dihydro-2’*H*,4’*H*,5*H*-spiro[pyrano[3,2-*c*]chromene-4,1’-pyrrolo[3,2,1-*ij*]quinoline]-3-carbonitrile) was obtained by the three-component cyclocondensation of 5,6-dihydro-4*H*-pyrrolo[3,2,1-*ij*]quinolin-1,2-diones **Ia** with malononitrile and 4-hydroxycoumarin according to our previously developed method [45] in the environment of ethanol and the presence of *N*-methylpiperazine as a catalyst (see Scheme 1).

**Scheme 2:**
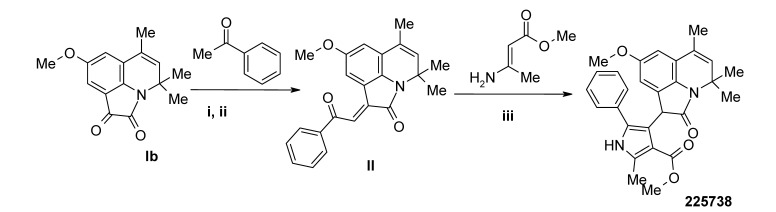
Synthetic route to methyl 4-(8-methoxy-4,4,6-trimethyl-2-oxo-1,2-dihydro-4*H*-pyrrolo[3,2,1-*ij*]quinolin-1-yl)-2-methyl-5-phenyl-1*H*-pyrrole-3-carboxylate (**225738**). (i) Et3N, EtOH, reflux > rt; (ii): AcOH, HCl, reflux; (iii) Et_3_N, EtOH, reflux.

Compound **225738** (methyl 4-(8-methoxy-4,4,6-trimethyl-2-oxo-1,2-dihydro-4*H*-pyrrolo[3,2,1-*ij*]quinolin-1-yl)-2-methyl-5-phenyl-1*H*-pyrrole-3-carboxylate) was first synthesized by the interaction of 1-phenacylidene-pyrroloquinolin-2-one **II** (obtained by condensation of pyrrolo[3,2,1-*ij*]quinoline-1,2-dione **Ib** with acetophenone according to our recently published method [46]) with methyl 3-aminocrotonate (see Scheme 2).

The presence of two asymmetric centers in the molecule of synthesized compound 30901 gave rise to the possibility of stereoisomers: the ^1^H NMR spectra contained two sets of signals. The molar ratio of components in the mixture was determined by comparing the integrated signal intensities of the respective protons in aromatic fragment and methyl and methylene groups of hydroquinoline ring system and amino group, and was in the range of 1:2. This was also confirmed by ^13^C NMR spectral data. Analogically, the product **225738** was obtained as a mixture of two rotamers.

#### 3.4.1. Instrumentation

NMR ^1^H and ^13^C spectra were registered on a Bruker DRX−500 (500.13 and 125.76 MHz, respectively) spectrometer (Bruker Corporation, Billerica, MA, USA) in DMSO-d6, and the internal standard was TMS. Melting points were determined on Stuart SMP 30 (Cole-Palmer, Staffordshire, UK). To control the reagent and product individuality, qualitative analysis of reaction mass was performed by TLC on Merck TLC Silicagel 60 F254 chromatographic plate (Merck KGaA, Darmstadt, Germany); eluents: methanol, chloroform, and their mixtures in various rations. The chromatograms were developed by UV (Irradiator of chromatographic plates UFS 254/365 Sorbfil, Production company Imid, Krasnodar, Russia) and iodine vapor. Product purity was monitored by high-performance liquid chromatography with high-resolution mass spectrometric electrospray ionization detection (HPLCHRMS–ESI) in combination with UV detection. The analyses were performed on an Agilent 1260 Infinity chromatograph (Agilent Technologies, Santa Clara, CA, USA) and Agilent 6230 TOF LC/MS high-resolution time-of-flight mass detector. The ionization block was double electrospray; the signals were recorded in positive polarity; nebulizer N2 20 psig; desiccant gas N2, 6 mL/min, 325 °C; mass detection range was 50–2000 daltons. Capillary voltage 4.0 kV, fragmentator +191 V, skimmer +66 V, OctRF 750 V. Poroshell 120 EC-C18 column (4.6 × 50 mm; 2.7 µm) was used. Gradient elution: acetonitrile/water (0.1% formic acid); flow rate 0.4 mL/min. Software for processing research results: MassHunter Workstation/Data Acquisition V.06.00 (Agilent Technologies, Santa Clara, CA, USA).

#### 3.4.2. Chemicals

The starting compound **Ia**,**b**, **II** was synthesized according to a published method [46,47]. Commercially available reagents from Lancaster were also used in the syntheses.

Procedure for the synthesis of 2-amino-8’-methoxy-4’,4’,6’-trimethyl-2’,5-dioxo-5’,6’-dihydro-2’*H*,4’*H*,5*H*-spiro[pyrano[3,2-*c*]chromene-4,1’-pyrrolo[3,2,1-*ij*]quinoline]-3-carbonitrile **30901**: a mixture of the pyrrolo[3,2,1-*ij*]quinoline-1,2-dione **Ia** (0.6 g, 2.5 mmol), malononitrile (0.17 g, 2.5 mmol), and 4-hydroxycoumarin (0.4 g, 2.5 mmol) in ethanol (10 mL) containing 2–3 drops of *N*-methylpiperazine as the catalyst was refluxed for 15 min and cooled. The obtained precipitate was filtered, dried, and crystallized from ethanol. White powder; 0.89 g; yield 76%; m.p. >300 °C; ^1^H NMR (500 MHz, DMSO-d_6_), δ (ppm): major diastereomer—1.31 (3H, s, CH_3_); 1.35 (3H;, s, (CH_3_)_2_); 1.48–1.54 (1H, m, CH_2_); 1.67 (3H, s, 6-(CH_3_)_2_); 1.87–1.92 (1H, m, CH_2_); 2.88–2.93 (1H, m, CH); 3.67 (3H, s, OCH_3_); 6.77 + 6.79 (2H, 2s, NH_2_); 7.48 (1H, d, *J* = 7.8 Hz, H Ar); 7.49 (1H, s, H-7′); 7.53 (1H, t, *J* = 7.8 Hz, H Ar); 7.63 (1H, s, H-9′); 7.77 (1H, t, *J* = 7.8 Hz, H Ar); 7.95 (1H, d, *J* = 7.8 Hz, H Ar); minor diastereomer—1.32 (3H, s, CH_3_); 1.34 (3H, s, (CH_3_)_2_); 1.52–1.57 (1H, m, CH_2_); 1.88–1.91 (1H, m, CH_2_); 3.68 (3H, s, OCH_3_); 6.78 (2H, 2s, NH_2_); 7.60 (1H, s, H-9′). ^13^C NMR (125 MHz, DMSO-d_6_), δ (ppm):18.28, 23.14, 24.75, 25.97, 26.63, 46.18, 53.70, 55.59, 57.11, 101.75, 108.28, 110.94, 112.62, 116.70, 122.71, 124.98, 125.24, 131.82, 132.98, 133.59, 152.15, 155.12, 155.86, 158.25, 158.38, 174.22; minor diastereomer—26.03, 26.59, 45.86, 54.00, 108.43, 116.82, 125.28, 131.66, 152.09, 155.07, 155.83, 158.30, 158.42. HPLC–HRMS–ESI, *m/z* ([M + H]^+^), calculated for C_27_H_23_N_3_O_5_ + H^+^ 470.1712, found as 470.1709 (see Supplementary Materials, Figures S1–S3).

Procedure for the synthesis of methyl 4-(8-methoxy-4,4,6-trimethyl-2-oxo-1,2- dihydro-4*H*-pyrrolo[3,2,1-*ij*]quinolin-1-yl)-2-methyl-5-phenyl-1*H*-pyrrole-3-carboxylate **225738**: a mixture of the 1-phenacylidene-pyrroloquinolin-2-one **II** (0.5 g, 1.5 mmol) and methyl 3-aminocrotonate (0.5 g, 1.5 mmol) in ethanol (10 mL) containing 1 mL Et_3_N as the catalyst was refluxed for 7 h and cooled. The obtained precipitate was filtered, dried, and crystallized from ethanol. White powder; 0.49 g; yield 72%; m.p. 212–214 °C; ^1^H NMR (500 MHz, DMSO-d_6_), δ (ppm): major rotamer—1.59 (3H, s, 4-(CH_3_)); 1.64 (3H, s, 4-(CH_3_)); 1.99 (3H, s, 6-(CH_3_)); 2.43 (3H, s, CH_3pyrrol_);3.26 (3H, s, OCH_3_); 3.65 (3H, s, OCH_3_); 4.53 (1H, s, H-1); 5.40 (1H, s, H-5); 6.33–6.34 (1H, m, H Ar); 6.58 (1H, s, H Ar); 7.35–7.38 (1H, m, H Ar); 7.46–7.50 (2H, m, H Ar); 7.54–7.57 (2H, m, H Ar); 11.60 (1H, s, NH); minor rotamer—1.14 (3H, s, 4-(CH_3_)); 1.48 (3H, s, 4-(CH_3_)); 1.96 (3H, s, 6-(CH_3_)); 2.53 (3H, s, CH_3pyrrol_); 3.62 (3H, s, OCH_3_); 3.77 (3H, s, OCH_3_); 5.28 (1H, s, H-1); 5.60 (1H, s, H-5); 6.41 (1H, s, H Ar); 6.77–6.78 (1H, m, H Ar); 7.08–7.15 (2H, m, H Ar); 7.35–7.38 (3H, m, H Ar); 11.44 (1H, s, NH). Two rotamers, 6:1 ratio. ^13^C NMR (125 MHz, DMSO-d_6_), δ (ppm): 13.25, 17.15, 26.97, 27.72, 45.50, 49.82, 55.68, 56.16, 106.20, 108.14, 109.52 113.65, 118.07, 124.85, 127.45, 127.82, 127.95, 128.40, 128.88, 130.88, 131.67, 132.07, 133.34, 136.36, 155.40, 164.44, 175.93. HPLC–HRMS–ESI, *m/z* ([M + H]^+^), calculated for C_28_H_28_N_2_O_4_ + H^+^ 457.2123, found as 457.2126 (see Supplementary Materials, Figures S4–S6).

HPLC–MS data of tested compounds:

2-Amino-8’-ethyl-4’,4’,6’-trimethyl-2’,5-dioxo-5,5’,6,6’-tetrahydro-4’*H*-spiro[pyrano[3,2-*c*]quinoline-4,1’-pyrrolo[3,2,1-*ij*]quinoline]-3-carbonitrile **38292,** HPLC–HRMS–ESI, *m/z* ([M + H]^+^), calculated for C_28_H_26_N_4_O_3_ + H^+^ 467.2079, found as 467.2077 (see Supplementary Materials, Figure S7).

2-Amino-4-(3,4-dimethoxyphenyl)-5-oxo-5,6-dihydro-4*H*-pyrano[3,2-*c*]quinoline-3-carbonitrile **38756**, HPLC–HRMS–ESI, *m/z* ([M + H]^+^), calculated for C_21_H_17_N_3_O_4_ + H^+^ 376.1293, found as 376.1296 (see Supplementary Materials, Figure S8).

6’,6’-Dimethyl-2,3,4,5’,6’,9-hexahydrodispiro[*β*-carboline-1,1’-pyrrolo[3,2,1-*ij*]quinoline-4’,1’’-cyclohexan]-2’-one **41453,** HPLC–HRMS–ESI, *m/z* ([M + H]^+^), calculated for C_28_H_31_N_3_O + H^+^ 426.2541, found as 426.2542 (see Supplementary Materials, Figure S9).

6-Methyl-2-phenyl-5,6,7,8-tetrahydrofuro[2,3-*b*]quinolin-4-amine **224530,** HPLC–HRMS–ESI, *m/z* ([M + H]^+^), calculated for C_18_H_18_N_2_O + H^+^ 279.1493, found as 279.1482 (see Supplementary Materials, Figure S10).

4-Amino-2-(2-chlorophenyl)-7,8-dihydrofuro[2,3-*b*]quinolin-5(6*H*)-one **224533**, HPLC–HRMS–ESI, *m/z* ([M + H]^+^), calculated for C_17_H_13_ClN_2_O_2_ + H^+^ 313.0739, found as 313.0737 (see Supplementary Materials, Figure S11).

2-(8-Methoxy-4,4,6-trimethyl-2-oxo-4*H*-pyrrolo[3,2,1-ij]quinolin-1(2*H*)-ylidene)hydrazinecarboximidamide **225635,** HPLC–HRMS–ESI, *m/z* ([M + H]^+^), calculated for C_16_H_19_N_5_O_2_ + H^+^ 314,1612, found as 314.1614 (see Supplementary Materials, Figure S12).

2-[6-{[4-(4-Fluorophenyl)piperazin-1-yl]methyl}-8-methoxy-4,4-dimethyl-2-oxo-4*H*-pyrrolo[3,2,1-*ij*]quinolin-1(2*H*)-ylidene]hydrazinecarboximidamide **225638,** HPLC–HRMS–ESI, *m/z* ([M + H]^+^), calculated for C_26_H_30_FN_7_O_2_ + H^+^ 492.2519, found as 492.2518 (see Supplementary Materials, Figure S13).

8-Methoxy-4,4,6-trimethyl-4*H*-pyrrolo[3,2,1-*ij*]quinoline-1,2-dione1-(*O*-benzoyloxime) **225746,** HPLC–HRMS–ESI, *m/z* ([M + H]^+^), calculated for C_22_H_20_N_2_O_4_ + H^+^ 377.1497, found as 377.1494 (see Supplementary Materials, Figure S14).

6,8,8,9-Tetramethyl-3-(2-morpholin-4-yl-6-oxo-1,6-dihydropyrimidin-4-yl)-6,7,8,9-tetrahydro-2*H*-pyrano[3,2-*g*]quinolin-2-one **225006,** HPLC–HRMS–ESI, *m/z* ([M + H]^+^), calculated for C_24_H_28_N_4_O_4_ + H^+^ 437,2185, found as 437.2185 (see Supplementary Materials, Figure S15).

3-(4-Methoxybenzoyl)-6,8,8,9-tetramethyl-6,7,8,9-tetrahydro-2*H*-pyrano[3,2-*g*]quinolin-2-one **224870**, HPLC–HRMS–ESI, *m/z* ([M + H]^+^), calculated for C_24_H_25_NO_4_ + H^+^ 392,1858, found as 392.1855 (see Supplementary Materials, Figure S16).

6-((4-Benzo[*d*][1,3]dioxol-5-ylmethyl)piperazine-1-yl)methyl)-8-fluoro-4,4-dimethyl–1*H*-pyrrolo[3,2,1-*ij*]quinolin-1,2(4*H*)-dione **225624**. HPLC–HRMS–ESI, *m/z* ([M + H]^+^), calculated for C_26_H_26_FN_3_O_4_ + H^+^ 464.1981, found as 464.1984. (see Supplementary Materials, Figure S17).

### 3.5. In Vitro Assay

The inhibition of factor XIIa by the synthesized compounds was studied by measuring the kinetics of hydrolysis of specific substrate S2302 (Chromogenics, Italy) in the presence of these compounds. A buffer containing 140 mM NaCl, 20 mM HEPES and 0.1% PEG 6000 (pH 7.4) was placed in the wells of a 96-well plate, followed by the addition of factor XIIa (final concentration 5 nM), solution of the test compound in DMSO (final concentration 30 μM), and substrate S2302 (Chromogenics, Italy) (final concentration 200 μM). The DMSO content in the well was no more than 2%. The kinetics of the formation of pNa were measured using a THERMOmax Microplate Reader (Molecular Devices Corporation, USA) by the absorption of light with a wavelength of 405 nm. The initial rate of substrate degradation was determined from the initial slope of the pNa formation curve. The rate of substrate degradation by the enzyme in the presence of the inhibitor was expressed as a percentage relative to the rate of substrate degradation in the absence of the inhibitor. The data obtained were processed using the GraphPad Prism and OriginPro 8 software. Ratio of specific substrate cleavage (S2302 for factor XIIa and S2765 for factor Xa) rate in presence of different inhibitor concentrations to these and the cleavage rate without inhibitor were determined to calculate IC_50_ values. Received curves (see Figures S18–S22) were approximated with the assumption that these were competitive inhibitors.

## 4. Conclusions

In this work, new synthetic low-molecular-weight coagulation factor XIIa inhibitors were discovered using a combination of molecular modeling, synthesis, and in vitro experimental validation. An atomistic model of the blood coagulation factor XIIa was built based on a three-dimensional structure from PDB (ID 6B74). To search for noncovalent inhibitors of factor XIIa, a focused library of 59 compounds selected from the database of druglike substances of the Department of Organic Chemistry of the Voronezh State University (VSU) was used. The search was carried out using the SOL docking program followed by the quantum chemical calculation of the protein–ligand binding enthalpy for the ligand poses in the active site of the protein found by docking. As a result of the calculations and visual analysis of the docked ligand poses, thirteen compounds were selected for in vitro testing to determine their inhibitory activity against the coagulation factor XIIa. As a result of in vitro testing, the inhibitory activity was confirmed for four compounds. One of them, **225006,** possessed low micromolar activity. Selectivity testing revealed that two of the identified factor XIIa inhibitors, **225006** and **225738**, were selective over the coagulation factors Xa and XIa. The findings of this study have to be seen in the light of some limitations. The main limitation is the size of the library that was subjected to virtual screening. Evidently, the application of large virtual libraries can give one an opportunity to identify more diverse hit series and probably offer a higher hit rate. We selected a narrowly focused library since it was already profiled for two off-targets which were relevant for the design of factor XIIa inhibitors. So, the usage of this library gave us the opportunity to acquire inhibition data for off-targets after the screening procedure and facilitated the hit identification stage.

The best inhibitor contains a tricyclic fragment in which the coumarin bicycle is fused with a piperidine ring. It is noteworthy that a simple coumarin fragment is found in inhibitors reported in the literature. Two of the four confirmed factor XIIa inhibitors belong to the chemical class of synthetic spiroheterocycles and have in their structures the pharmacophoric fragment of pyrrolo[3,2,1-*ij*] quinolin-2-one, which forms a spiro system with fragments of the natural alkaloid *β*-carboline (at position one) and cyclohexane (according to position four). Obviously, the presence of an additional spiro fragment further increases the rigidity of the spatial structure and makes it more complementary to the three-dimensional binding sites of the most important biotargets (enzymes, receptors, and ion channels) [48,49,50,51].

The hit series found in this study can be applied for further structural optimization processes to increase their potency against factor XIIa.

## Figures and Tables

**Table 1:** Characteristics of the best ligands selected for experiments. ID is a compound number in the VSU library, SOL is a value of the SOL scoring function, ΔH_bind_ is the protein–ligand binding enthalpy calculated using the PM7+COSMO method, inhibition activity is the % of enzyme inhibition at 30 µM averaged over three measurements. ClogP and TPSA values were calculated with Chemaxon Marvin [32].

ID	Inhibition Activity at 30 µM, %	SOL, kcal/mol	ΔH_bind_, kcal/mol	ClogP	TPSA
**41453**	83	−7.03	−43.8	5.06	48.1
**30901**	72	−5.24	−34.5	2.37	114.9
**225738**	69	−5.11	−37.9	4.66	71.6
**225006**	50	−6.55	−15.5	2.37	83.5
**225638**	27	−5.39	−70.0	2.61	110.3
**38756**	15	−5.12	−35.2	1.74	106.6
**38292**	10	−5.36	−36.6	3.30	108.5
**224533**	2	−5.28	−42.1	3.46	69.1
**224870**	0	−6.52	−30.4	4.54	55.8
**225624**	0	−6.19	−33.9	2.94	62.3
**225635**	0	−5.01	−69.0	1.30	103.8
**224530**	0	−5.01	−27.3	3.63	52.1
**225746**	0	−5.44	−38.4	4.22	68.2

## Data Availability

Not applicable.

## Supplementary Materials

The following supporting information on the NMR spectra and data of the HPLC-MS-ESI analysis of compounds **30901** and **225738** and the data of the HPLC-MS-ESI analysis of compounds **225638**, **225635**, **41453**, **224533**, **225746**, **225006**, **225624**, **224870**, **224530**, **38292**, and **38756** can be downloaded at online, Figure S1: NMR ^13^C spectra of 2-amino-8’-methoxy-4’,4’,6’-trimethyl-2’,5-dioxo-5’,6’-dihydro-2’H,4’H,5H-spiro[pyrano[3,2-c]chromene-4,1’-pyrrolo[3,2,1-ij]quinoline]-3-carbonitrile **30901**, Figure S2: NMR ^1^H spectra of 2-amino-8’-methoxy-4’,4’,6’-trimethyl-2’,5-dioxo-5’,6’-dihydro-2’H,4’H,5H-spiro[pyrano[3,2-c]chromene-4,1’-pyrrolo[3,2,1-ij]quinoline]-3-carbonitrile **30901**, Figure S3: HPLC–HRMS–ESI spectra of 2-amino-8’-methoxy-4’,4’,6’-trimethyl-2’,5-dioxo-5’,6’-dihydro-2’H,4’H,5H-spiro[pyrano[3,2-c]chromene-4,1’-pyrrolo[3,2,1-ij]quinoline]-3-carbonitrile **30901**, Figure S4: NMR ^13^C spectra of methyl 4-(8-methoxy-4,4,6-trimethyl-2-oxo-1,2-dihydro-4*H*-pyrrolo[3,2,1-*ij*]quinolin-1-yl)-2-methyl-5-phenyl-1*H*-pyrrole-3-carboxylate **225738**, Figure S5: NMR ^1^H spectra of methyl 4-(8-methoxy-4,4,6-trimethyl-2-oxo-1,2-dihydro-4*H*-pyrrolo[3,2,1-*ij*]quinolin-1-yl)-2-methyl-5-phenyl-1*H*-pyrrole-3-carboxylate **225738**, Figure S6: HPLC–HRMS–ESI spectra of methyl 4-(8-methoxy-4,4,6-trimethyl-2-oxo-1,2-dihydro-4*H*-pyrrolo[3,2,1-*ij*]quinolin-1-yl)-2-methyl-5-phenyl-1*H*-pyrrole-3-carboxylate **225738**, Figure S7: HPLC–HRMS–ESI spectra of 2-amino-8’-ethyl-4’,4’,6’-trimethyl-2’,5-dioxo-5,5’,6,6’-tetrahydro-4’*H*-spiro[pyrano[3,2-*c*]quinoline-4,1’-pyrrolo[3,2,1-*ij*]quinoline]-3-carbonitrile **38292**, Figure S8: HPLC–HRMS–ESI spectra of 2-amino-4-(3,4-dimethoxyphenyl)-5-oxo-5,6-dihydro-4*H*-pyrano[3,2-*c*]quinoline-3-carbonitrile **38756**, Figure S9: HPLC–HRMS–ESI spectra of 6’,6’-dimethyl-2,3,4,5’,6’,9-hexahydrodispiro[*β*-carboline-1,1’-pyrrolo[3,2,1-*ij*]quinoline-4’,1’’-cyclohexan]-2’-one **41453,** Figure S10: HPLC–HRMS–ESI spectra of 6-methyl-2-phenyl-5,6,7,8-tetrahydrofuro[2,3-*b*]quinolin-4-amine **224530**, Figure S11: HPLC–HRMS–ESI spectra of 4-amino-2-(2-chlorophenyl)-7,8-dihydrofuro[2,3-*b*]quinolin-5(6*H*)-one **224533**, Figure S12: HPLC–HRMS–ESI spectra of 2-(8-methoxy-4,4,6-trimethyl-2-oxo-4*H*-pyrrolo[3,2,1-*ij*]quinolin-1(2*H*)-ylidene)hydrazinecarboximidamide **225635**, Figure S13: HPLC–HRMS–ESI spectra of 2-[6-{[4-(4-fluorophenyl)piperazin-1-yl]methyl}-8-methoxy-4,4-dimethyl-2-oxo-4*H*-pyrrolo[3,2,1-*ij*]quinolin-1(2*H*)-ylidene]hydrazinecarboximidamide **225638**. Figure S14: HPLC–HRMS–ESI spectra of 8-methoxy-4,4,6-trimethyl-4*H*-pyrrolo[3,2,1-*ij*]quinoline-1,2-dione1-(*O*-benzoyloxime) **225746**, Figure S15: HPLC–HRMS–ESI spectra of 6,8,8,9-tetramethyl-3-(2-morpholin-4-yl-6-oxo-1,6-dihydropyrimidin-4-yl)-6,7,8,9-tetrahydro-2*H*-pyrano[3,2-*g*]quinolin-2-one **225006**, Figure S16: HPLC–HRMS–ESI spectra of 3-(4-methoxybenzoyl)-6,8,8,9-tetramethyl-6,7,8,9-tetrahydro-2*H*-pyrano[3,2-*g*]quinolin-2-one **224870**, Figure S17: HPLC–HRMS–ESI spectra of 6-((4-benzo[d][1,3]dioxol-5-ylmethyl)piperazine-1-yl)methyl)-8-fluoro-4,4-dimethyl–1*H*-pyrrolo[3,2,1-*ij*]quinolin-1,2(4*H*)-dione **225624**.

## Sample Availability

Samples of the compounds 30901, 41453, 225738, and 225006 are available from the authors.

## References

[1] Xu D., Xue G., Peng B., Feng Z., Hongling L., Gong L. (2020). High-Throughput Docking and Molecular Dynamics Simulations towards the Identification of Potential Inhibitors against Human Coagulation Factor XIIa. Comput. Math. Methods Med..

[2] Podoplelova N.A., Sulimov V.B., Tashchilova A.S., Ilin I.S., Panteleev M.A., Ledeneva I.V., Shikhaliev K.S. (2020). Blood coagulation in the 21st cetrury: Existing knowledge, current strategies for treatment and perspective. Pediatr. Hematol. Immunopathol..

[3] Kleinschnitz C., Stoll G., Bendszus M., Schuh K., Pauer H.-U., Burfeind P., Renneé C., Gailani D., Nieswandt B., Renneé T. (2006). Targeting coagulation factor XII provides protection from pathological thrombosis in cerebral ischemia without interfering with hemostasis. J. Exp. Med..

[4] Müller F., Gailani D., Renné T. (2011). Factor XI and XII as antithrombotic targets. Curr. Opin. Hematol..

[5] Hagedorn I., Schmidbauer S., Pleines I., Kleinschnitz C., Kronthaler U., Stoll G., Dickneite G., Nieswandt B. (2010). Factor XIIa Inhibitor Recombinant Human Albumin Infestin-4 Abolishes Occlusive Arterial Thrombus Formation Without Affecting Bleeding. Circulation.

[6] Cheng Q., Tucker E.I., Pine M.S., Sisler I., Matafonov A., Sun M., White-Adams T.C., Smith S.A., Hanson S.R., McCarty O.J.T. (2010). A role for factor XIIa–mediated factor XI activation in thrombus formation in vivo. Blood.

[7] Xu Y., Cai T.-Q., Castriota G., Zhou Y., Hoos L., Jochnowitz N., Loewrigkeit C., Cook J.A., Wickham A., Metzger J.M. (2014). Factor XIIa inhibition by Infestin-4: In vitro mode of action and in vivo antithrombotic benefit. Thromb Haemost.

[8] Matafonov A., Leung P.Y., Gailani A.E., Grach S.L., Puy C., Cheng Q., Sun M., McCarty O.J.T., Tucker E.I., Kataoka H. (2014). Factor XII inhibition reduces thrombus formation in a primate thrombosis model. Blood.

[9] Pathak M., Wilmann P., Awford J., Li C., Hamad B.K., Fischer P.M., Dreveny I., Dekker L.V., Emsley J. (2015). Coagulation factor XII protease domain crystal structure. J. Thromb. Haemost..

[10] Cool D.E., Edgell C.J., Louie G.V., Zoller M.J., Brayer G.D., MacGillivray R.T. (1985). Characterization of human blood coagulation factor XII cDNA. Prediction of the primary structure of factor XII and the tertiary structure of beta-factor XIIa. J. Biol. Chem..

[11] Dementiev A., Silva A., Yee C., Li Z., Flavin M.T., Sham H., Partridge J.R. (2018). Structures of human plasma β–factor XIIa cocrystallized with potent inhibitors. Blood Adv..

[12] Larsson M., Rayzman V., Nolte M.W., Nickel K.F., Björkqvist J., Jämsä A., Hardy M.P., Fries M., Schmidbauer S., Hedenqvist P. (2014). A Factor XIIa Inhibitory Antibody Provides Thromboprotection in Extracorporeal Circulation Without Increasing Bleeding Risk. Sci. Transl. Med..

[13] Baeriswyl V., Calzavarini S., Chen S., Zorzi A., Bologna L., Angelillo-Scherrer A., Heinis C. (2015). A Synthetic Factor XIIa Inhibitor Blocks Selectively Intrinsic Coagulation Initiation. ACS Chem. Biol..

[14] Woodruff R.S., Xu Y., Layzer J., Wu W., Ogletree M.L., Sullenger B.A. (2013). Inhibiting the intrinsic pathway of coagulation with a factor XII-targeting RNA aptamer. J. Thromb. Haemost..

[15] Cai T.-Q., Wu W., Shin M.K., Xu Y., Jochnowitz N., Zhou Y., Hoos L., Bentley R., Strapps W., Thankappan A. (2015). Factor XII full and partial null in rat confers robust antithrombotic efficacy with no bleeding. Blood Coagul. Fibrinolysis.

[16] Revenko A.S., Gao D., Crosby J.R., Bhattacharjee G., Zhao C., May C., Gailani D., Monia B.P., MacLeod A.R. (2011). Selective depletion of plasma prekallikrein or coagulation factor XII inhibits thrombosis in mice without increased risk of bleeding. Blood.

[17] Davoine C., Bouckaert C., Fillet M., Pochet L. (2020). Factor XII/XIIa inhibitors: Their discovery, development, and potential indications. Eur. J. Med. Chem..

[18] Bouckaert C., Serra S., Rondelet G., Dolušić E., Wouters J., Dogné J.-M., Frédérick R., Pochet L. (2016). Synthesis, evaluation and structure-activity relationship of new 3-carboxamide coumarins as FXIIa inhibitors. Eur. J. Med. Chem..

[19] Bouckaert C., Zhu S., Govers-Riemslag J.W.P., Depoorter M., Diamond S.L., Pochet L. (2017). Discovery and assessment of water soluble coumarins as inhibitors of the coagulation contact pathway. Thromb. Res..

[20] Chen J.J.F., Visco D.P. (2017). Identifying novel factor XIIa inhibitors with PCA-GA-SVM developed vHTS models. Eur. J. Med. Chem..

[21] Platte S., Korff M., Imberg L., Balicioglu I., Erbacher C., Will J.M., Daniliuc C.G., Karst U., Kalinin D.V. (2021). Microscale Parallel Synthesis of Acylated Aminotriazoles Enabling the Development of Factor XIIa and Thrombin Inhibitors. ChemMedChem.

[22] Korff M., Imberg L., Will J.M., Bückreiß N., Kalinina S.A., Wenzel B.M., Kastner G.A., Daniliuc C.G., Barth M., Ovsepyan R.A. (2020). Acylated 1H-1,2,4-Triazol-5-amines Targeting Human Coagulation Factor XIIa and Thrombin: Conventional and Microscale Synthesis, Anticoagulant Properties, and Mechanism of Action. J. Med. Chem..

[23] Sulimov A.V., Kutov D.C., Oferkin I.V., Katkova E.V., Sulimov V.B. (2013). Application of the docking program SOL for CSAR benchmark. J. Chem. Inf. Model..

[24] Sulimov V.B., Ilin I.S., Kutov D.C., Sulimov A. (2020). V Development of docking programs for Lomonosov supercomputer. J. Turkish Chem. Soc. Sect. A Chem..

[25] Stewart J.J. (2013). Optimization of parameters for semiempirical methods VI: More modifications to the NDDO approximations and re-optimization of parameters. J. Mol. Model..

[26] Klamt A., Schuurmann G. (1993). COSMO: A new approach to dielectric screening in solvents with explicit expressions for the screening energy and its gradient. J. Chem. Soc. Perkin Trans. 2.

[27] Stewart J.J.P. Stewart Computational Chemistry. MOPAC2016. http://openmopac.net/MOPAC2016.html.

[28] Berman H.M., Westbrook J., Feng Z., Gilliland G., Bhat T.N., Weissig H., Shindyalov I.N., Bourne P.E. (2000). The Protein Data Bank. Nucleic Acids Res..

[29] Lipinski C.A., Lombardo F., Dominy B.W., Feeney P.J. (2001). Experimental and computational approaches to estimate solubility and permeability in drug discovery and development settings. Adv. Drug Deliv. Rev..

[30] Veber D.F., Johnson S.R., Cheng H.-Y., Smith B.R., Ward K.W., Kopple K.D. (2002). Molecular properties that influence the oral bioavailability of drug candidates. J. Med. Chem..

[31] Hughes J.D., Blagg J., Price D.A., Bailey S., Decrescenzo G.A., Devraj R.V., Ellsworth E., Fobian Y.M., Gibbs M.E., Gilles R.W. (2008). Physiochemical drug properties associated with in vivo toxicological outcomes. Bioorg. Med. Chem. Lett..

[32] (2021). Marvin Was Used for Drawing, Displaying and Characterizing Chemical Structures, Substructures and Reactions, Marvin 21.3.0. https://chemaxon.com/products/marvin.

[33] DeLano W.L. (2002). The PyMOL Molecular Graphics System.

[34] Salentin S., Schreiber S., Haupt V.J., Adasme M.F., Schroeder M. (2015). PLIP: Fully automated protein-ligand interaction profiler. Nucleic Acids Res..

[35] Kutov D.C., Katkova E.V., Kondakova O.A., Sulimov A.V., Sulimov V.B. (2017). Influence of the method of hydrogen atoms incorporation into the target protein on the protein-ligand binding energy. Bull. South Ural State Univ. Ser. Math. Model. Program. Comput. Softw..

[36] Halgren T.A. (1996). Merck molecular force field. J. Comput. Chem..

[37] Sinauridze E.I., Romanov A.N., Gribkova I.V., Kondakova O.A., Surov S.S., Gorbatenko A.S., Butylin A.A., Monakov M.Y., Bogolyubov A.A., Kuznetsov Y.V. (2011). New synthetic thrombin inhibitors: Molecular design and experimental verification. PLoS ONE.

[38] Sulimov V.B., Katkova E.V., Oferkin I.V., Sulimov A.V., Romanov A.N., Roschin A.I., Beloglazova I.B., Plekhanova O.S., Tkachuk V.A., Sadovnichiy V.A. (2014). Application of molecular modeling to urokinase inhibitors development. Biomed Res. Int..

[39] Beloglazova I.B., Plekhanova O.S., Katkova E.V., Rysenkova K.D., Stambol’skii D.V., Sulimov V.B., Tkachuk V.A. (2015). Molecular modeling as a new approach to the development of urokinase inhibitors. Bull. Exp. Biol. Med..

[40] Sulimov V.B., Gribkova I.V., Kochugaeva M.P., Katkova E.V., Sulimov A.V., Kutov D.C., Shikhaliev K.S., Medvedeva S.M., Krysin M.Y., Sinauridze E.I. (2015). Application of molecular modeling to development of new factor Xa inhibitors. Biomed Res. Int..

[41] Novichikhina N., Ilin I., Tashchilova A., Sulimov A., Kutov D., Ledenyova I., Krysin M., Shikhaliev K., Gantseva A., Gantseva E. (2020). Synthesis, docking, and in vitro anticoagulant activity assay of hybrid derivatives of pyrrolo[3,2,1-ij]quinolin-2(1H)-one as new inhibitors of factor Xa and factor XIa. Molecules.

[42] Ilin I.S., Lipets E.N., Sulimov A.V., Kutov D.C., Shikhaliev K.S., Potapov A.Y., Krysin M.Y., Zubkov F.I., Sapronova L.V., Ataullakhanov F.I. (2019). New factor Xa inhibitors based on 1,2,3,4-tetrahydroquinoline developed by molecular modelling. J. Mol. Graph. Model..

[43] Medvedeva S.M., Krysin M.Y., Zubkov F.I., Nikitina E.V., Shikhaliev K.S. (2014). New Heterocyclic Systems Based on Substituted 3,4-Dihydro-1H-Spiro[Quinoline-2,1’-Cycloalkanes]. Chem. Heterocycl. Compd..

[44] Potapov A.Y., Paponov B.V., Podoplelova N.A., Panteleev M.A., Potapov M.A., Ledenyova I.V., Stolpovskaya N.V., Shikhaliev K.S. (2021). Synthesis of 2H-pyrano[3,2-g]quinolin-2-ones containing a pyrimidinone moiety and characterization of their anticoagulant activity via inhibition of blood coagulation factors Xa and XIa. Chem. Heterocycl. Compd..

[45] Medvedeva S.M., Sabynin A.L., Shikhaliev K.S. (2014). Efficient methods for the synthesis of spiroheterocyclic systems based on 4,4,6-trimethyl-4H-pyrrolo[3,2,1-ij]quinoline-1,2-diones. Russ. Chem. Bull..

[46] Novichikhina N.P., Skoptsova A.A., Shestakov A.S., Potapov A.Y., Kosheleva E.A., Kozaderov O.A., Ledenyova I.V., Podoplelova N.A., Panteleev M.A., Shikhaliev K.S. (2020). Synthesis and Anticoagulant Activity of New Ethylidene and Spiro Derivatives of Pyrrolo[3,2,1-ij]quinolin-2-ones. Russ. J. Org. Chem..

[47] Lescheva E., Medvedeva S., Shikhaliev K. (2014). Synthesis of 4,4,6-trimethyl-8-R-4H-pyrrolo [3,2,1-ij] quinoline-1,2-diones. Žurnal organìčnoï ta Farm. hìmìï.

[48] Galliford C.V., Scheidt K.A. (2007). Pyrrolidinyl-Spirooxindole Natural Products as Inspirations for the Development of Potential Therapeutic Agents. Angew. Chemie Int. Ed..

[49] Marti C., Carreira E.M. (2003). Construction of Spiro[pyrrolidine-3,3′-oxindoles]—Recent Applications to the Synthesis of Oxindole Alkaloids. European J. Org. Chem..

[50] Williams R.M., Cox R.J. (2003). Paraherquamides, Brevianamides, and Asperparalines:  Laboratory Synthesis and Biosynthesis. An Interim Report. Acc. Chem. Res..

[51] Alper P.B., Meyers C., Lerchner A., Siegel D.R., Carreira E.M. (1999). Facile, novel methodology for the synthesis of spiro [pyrrolidin-3,3′-oxindoles]: Catalyzed ring expansion reactions of cyclopropanes by aldimines. Angew. Chemie Int. Ed..

[52] Voevodin V.V., Antonov A.S., Nikitenko D.A., Shvets P.A., Sobolev S.I., Sidorov I.Y., Stefanov K.S., Voevodin V.V., Zhumatiy S.A. (2019). Supercomputer Lomonosov-2: Large scale, deep monitoring and fine analytics for the user community. Supercomput. Front. Innov..

