# Supplementary material for: New Blood Coagulation Factor XIIa Inhibitors: Molecular Modeling, Synthesis, and Experimental Confirmation
Source: Molecules. 2022 Feb 12;27(4):1234. doi: 10.3390/molecules27041234 (PMC8876603; doi:10.3390/molecules27041234)
Supplement: Supplementary file 1 [file molecules-27-01234-s001.zip › molecules-1571825-supplementary.pdf]

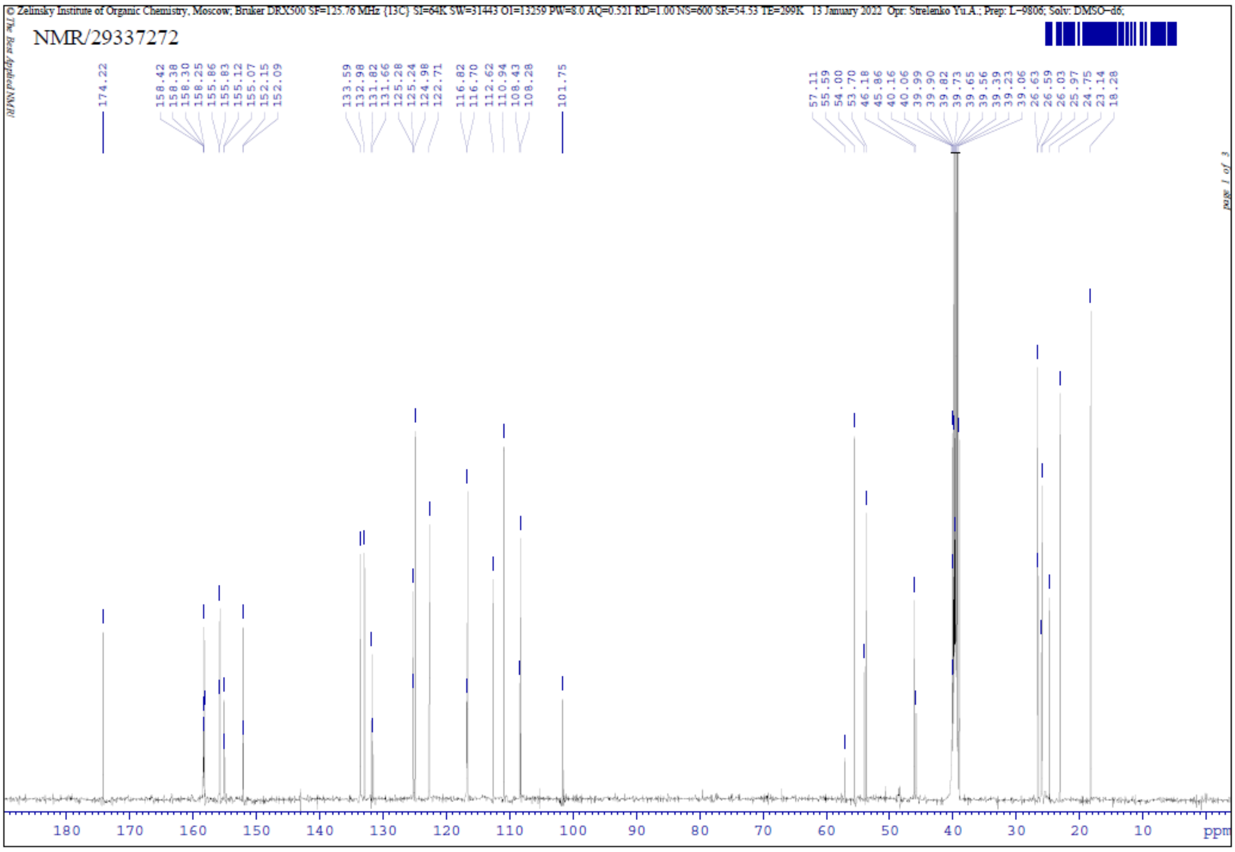

Figure S1: NMR ^{13}C spectra of 2-amino-8'-methoxy-4',4',6'-trimethyl-2',5-dioxo-5',6'-dihydro-2'*H*,4'*H*,5*H*-spiro[pyrano[3,2-*c*]chromene-4,1'-pyrrolo[3,2,1-*ij*]quinoline]-3-carbonitrile **30901.**

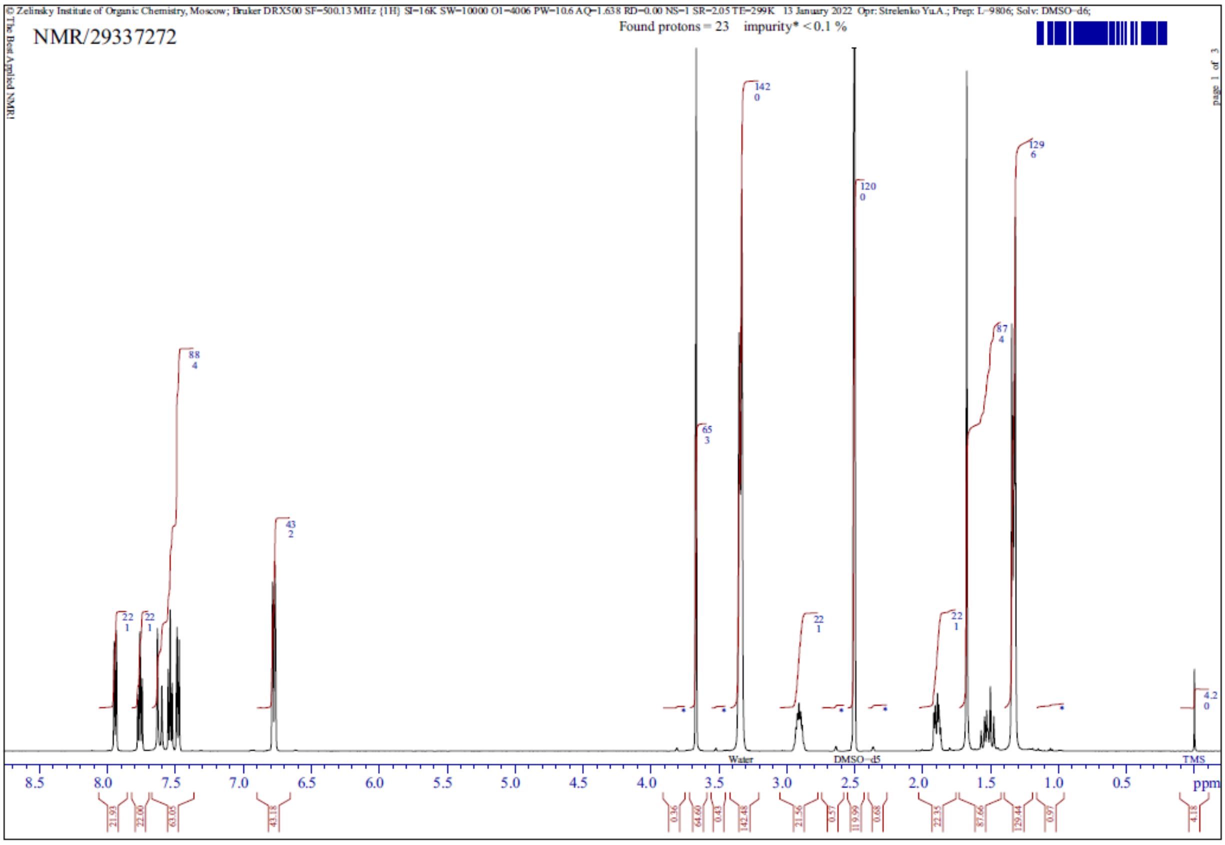

Figure S2: NMR ^{1}H spectra of 2-amino-8'-methoxy-4',4',6'-trimethyl-2',5-dioxo-5',6'-dihydro-2'*H*,4'*H*,5*H*-spiro[pyrano[3,2-*c*]chromene-4,1'-pyrrolo[3,2,1-*ij*]quinoline]-3-carbonitrile **30901.**

**Data Filename**	2329.d	**Sample Name**	
**Sample Type**	Sample	**Position**	Vial 78
**Instrument Name**	Instrument 1	**User Name**	
**Acq Method**	ACN-H2O_60-40.m	**Acquired Time**	12/30/2021 3:25:00 PM
**IRM Calibration Status**	Success	**DA Method**	111.m
**Comment**			

**Sample Group**		**Info.**	
**Stream Name**	LC 1	**Acquisition SW Version**	6200 series TOF/6500 series Q-TOF B.06.01 (B6172 SP1)

User Chromatograms

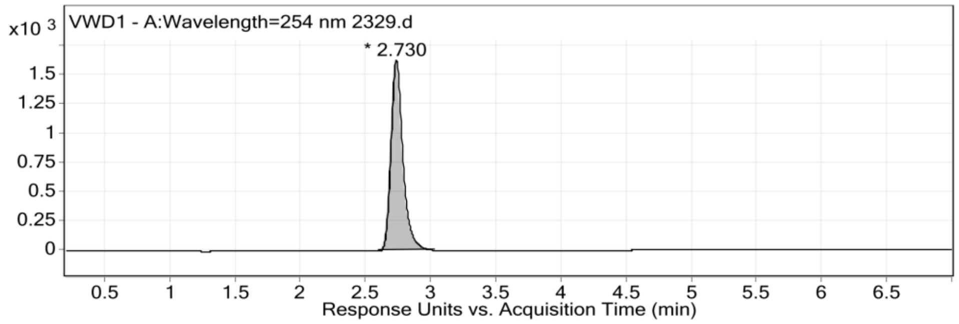

Integration Peak List

Peak	Start	RT	End	Height	Area	Area %
1	2.597	2.73	3.033	1627.09	10317.11	100

User Spectra

Fragmentor Voltage	Collision Energy	Ionization Mode
191	0	ESI

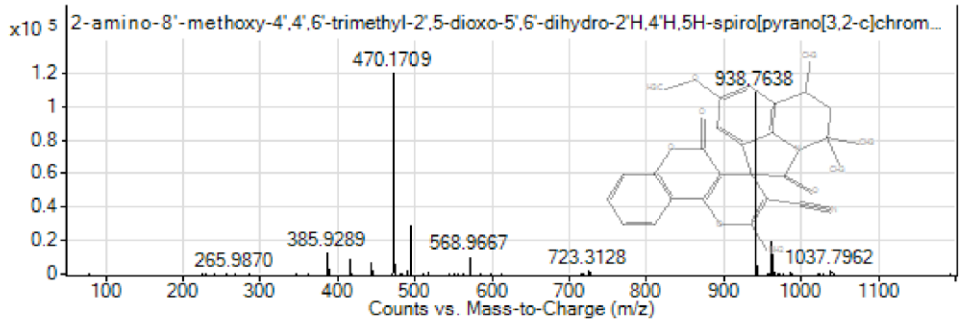

Figure S3: HPLC-HRMS-ESI spectra of 2-amino-8'-methoxy-4',4',6'-trimethyl-2',5-dioxo-5',6'-dihydro-2'*H*,4'*H*,5*H*-spiro[pyrano[3,2-*c*]chromene-4,1'-pyrrolo[3,2,1-*ij*]quinoline]-3-carbonitrile **30901.**

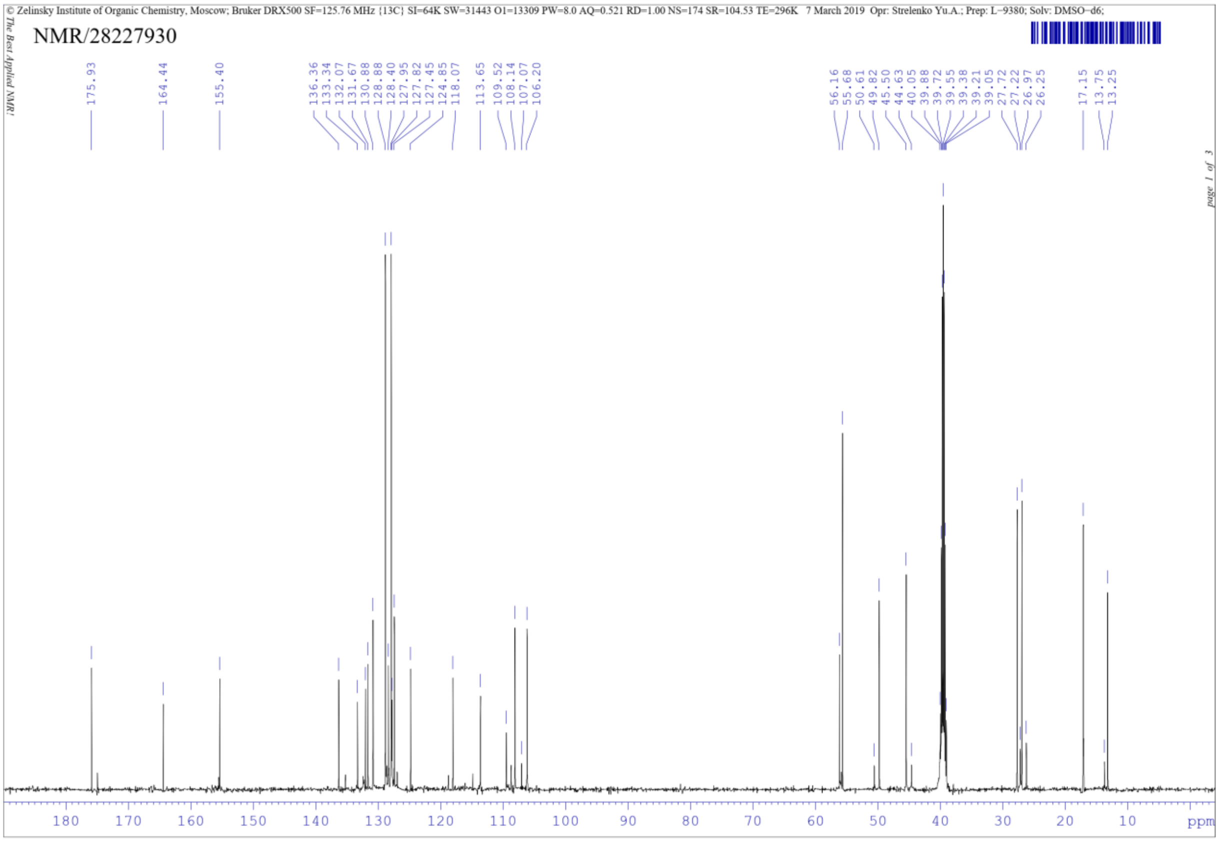

Figure S4: NMR ^{13}C spectra of methyl 4-(8-methoxy-4,4,6-trimethyl-2-oxo-1,2-dihydro-4*H*-pyrrolo[3,2,1-*ij*]quinolin-1-yl)-2-methyl-5-phenyl-1*H*-pyrrole-3-carboxylate **225738.**

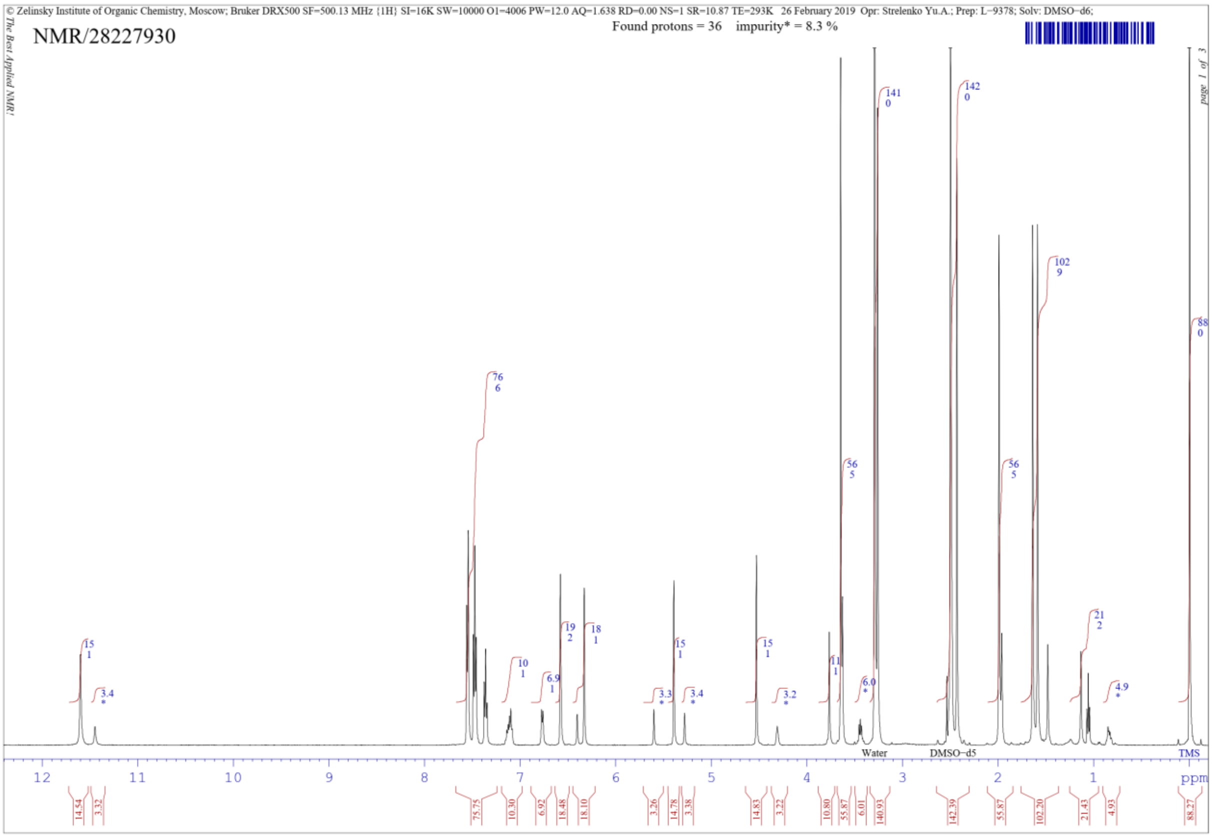

Figure S5: NMR ^{1}H spectra of methyl 4-(8-methoxy-4,4,6-trimethyl-2-oxo-1,2-dihydro-4*H*-pyrrolo[3,2,1-*ij*]quinolin-1-yl)-2-methyl-5-phenyl-1*H*-pyrrole-3-carboxylate **225738.**

**Data File**	LCMS_4687.d	**Sample Name**	
**Sample Type**	Sample	**Position**	Vial 19
**Instrument Name**	Instrument 1	**User Name**	
**Acq Method**	ACN-H2O_60-40.m	**Acquired Time**	19-Feb-19 12:04:32 PM
**IRM Calibration Status**	Success	**DA Method**	13032017.m
**Comment**			

**Sample Group**		**Info.**	
**Stream Name**	LC 1	**Acquisition SW Version**	6200 series TOF/6500 series Q-TOF B.06.01 (B6157)

Fragmentor Voltage 191 **Collision Energy** 0 **Ionization Mode** ESI

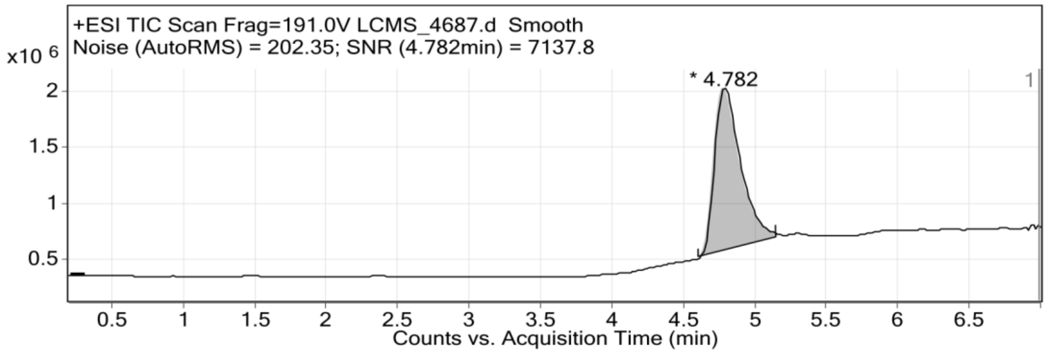

User Chromatogram Peak List

RT	Height	Height %	Area	Area %	Area Sum %	S/N	Symmetry	Width
4.782	1444357.36	100	18868633.4	100	100	7137.8	2.88	0.547

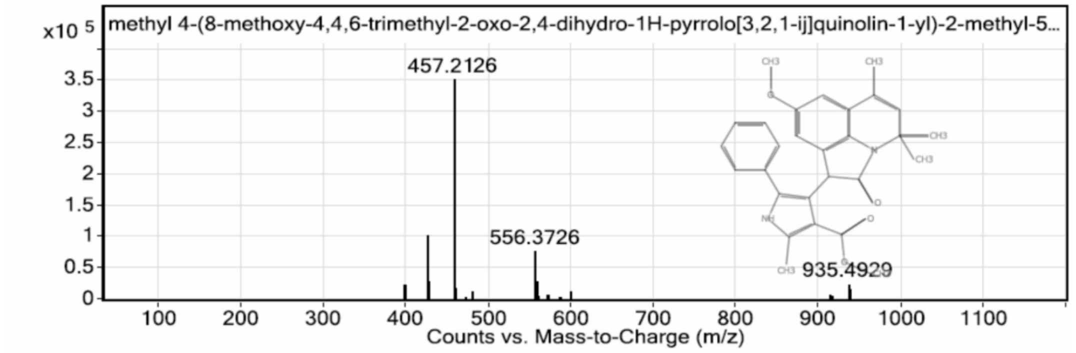

Figure S6: HPLC-HRMS-ESI spectra of methyl 4-(8-methoxy-4,4,6-trimethyl-2-oxo-1,2-dihydro-4*H*-pyrrolo[3,2,1-*ij*]quinolin-1-yl)-2-methyl-5-phenyl-1*H*-pyrrole-3-carboxylate **225738.**

Qualitative Analysis Report

**Data Filename**	2328.d	**Sample Name**	
**Sample Type**	Sample	**Position**	Vial 77
**Instrument Name**	Instrument 1	**User Name**	
**Acq Method**	ACN-H2O_60-40.m	**Acquired Time**	12/30/2021 3:13:01 PM
**IRM Calibration Status**	Success	**DA Method**	111.m
**Comment**			

**Sample Group**		**Info.**	
**Stream Name**	LC 1	**Acquisition SW Version**	6200 series TOF/6500 series Q-TOF B.06.01 (B6172 SP1)

User Chromatograms

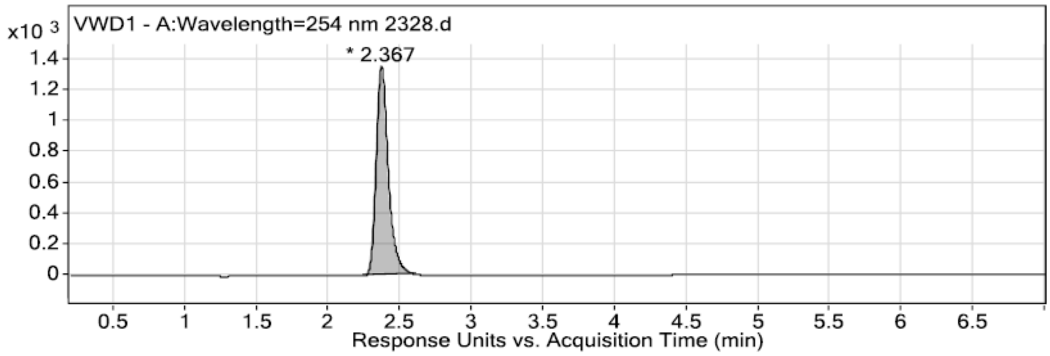

Integration Peak List

Peak	Start	RT	End	Height	Area	Area %
1	2.243	2.367	2.617	1358.78	7938.81	100

User Spectra

Fragmentor Voltage	Collision Energy	Ionization Mode
191	0	ESI

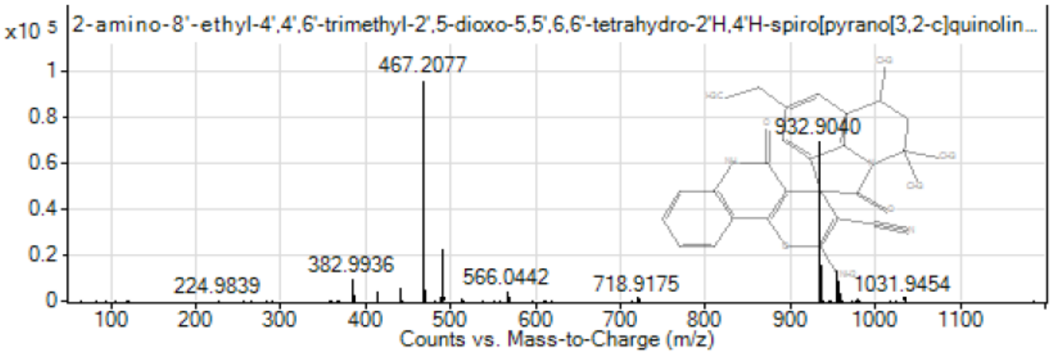

Figure S7: HPLC-HRMS-ESI spectra of 2-amino-8'-ethyl-4',4',6'-trimethyl-2',5-dioxo-5,5',6,6'-tetrahydro-4'*H*-spiro[pyrano[3,2-*c*]quinoline-4,1'-pyrrolo[3,2,1-*ij*]quinoline]-3-carbonitrile **38292.**

Qualitative Analysis Report

**Data Filename**	2322.d	**Sample Name**	
**Sample Type**	Sample	**Position**	Vial 71
**Instrument Name**	Instrument 1	**User Name**	
**Acq Method**	ACN-H2O_60-40.m	**Acquired Time**	12/30/2021 1:26:59 PM
**IRM Calibration Status**	Success	**DA Method**	111.m
**Comment**			

**Sample Group**		**Info.**	
**Stream Name**	LC 1	**Acquisition SW**	6200 series TOF/6500 series
		**Version**	Q-TOF B.06.01 (B6172 SP1)

User Chromatograms

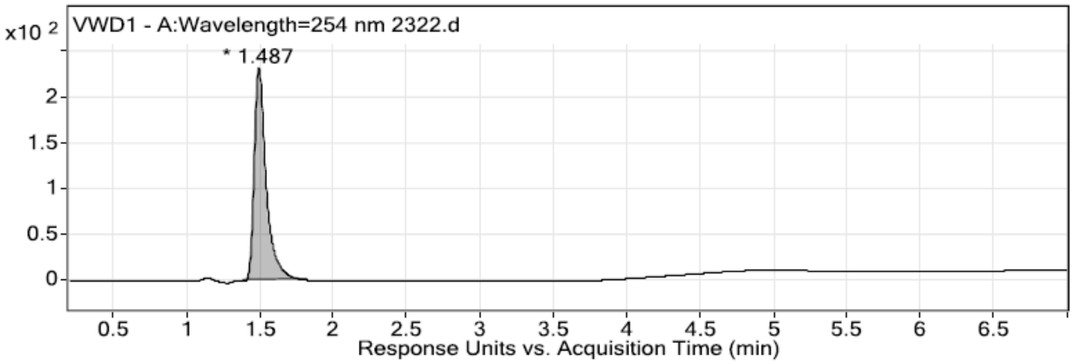

Integration Peak List

Peak	Start	RT	End	Height	Area	Area %
1	1.383	1.487	1.823	232.89	1316.06	100

User Spectra

Fragmentor Voltage	Collision Energy	Ionization Mode
191	0	ESI

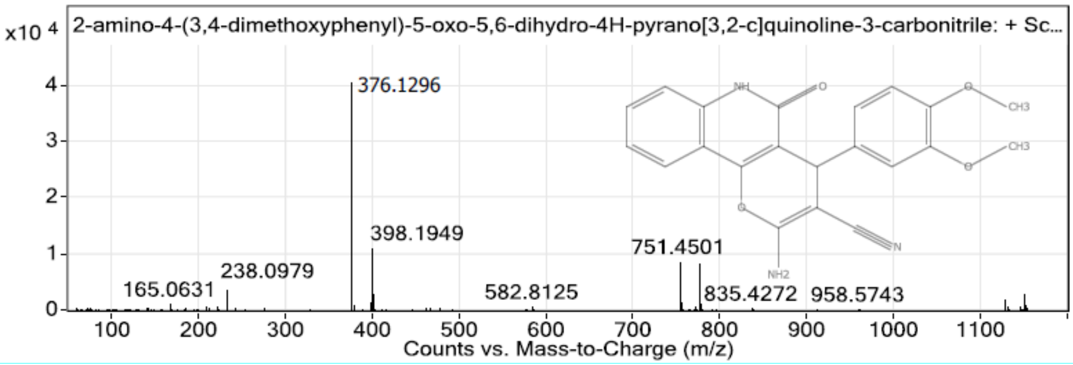

Figure S8: HPLC-HRMS-ESI spectra of 2-amino-4-(3,4-dimethoxyphenyl)-5-oxo-5,6-dihydro-4*H*-pyrano[3,2-*c*]quinoline-3-carbonitrile **38756.**

Qualitative Analysis Report

**Data Filename**	2325.d	**Sample Name**	
**Sample Type**	Sample	**Position**	Vial 74
**Instrument Name**	Instrument 1	**User Name**	
**Acq Method**	ACN-H2O_60-40.m	**Acquired Time**	12/30/2021 2:02:42 PM
**IRM Calibration Status**	Success	**DA Method**	111.m
**Comment**			

**Sample Group**		**Info.**	
**Stream Name**	LC 1	**Acquisition SW Version**	6200 series TOF/6500 series Q-TOF B.06.01 (B6172 SP1)

User Chromatograms

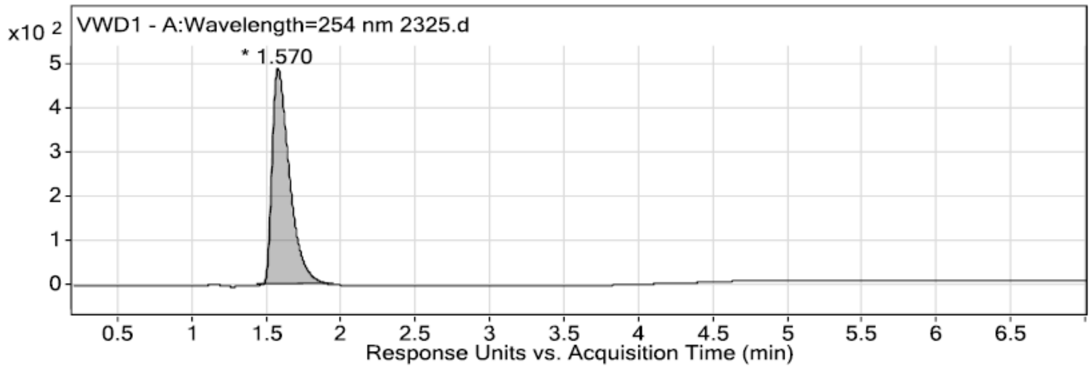

Integration Peak List

Peak	Start	RT	End	Height	Area	Area %
1	1.433	1.57	1.95	489.84	3976.52	100

User Spectra

Fragmentor Voltage	Collision Energy	Ionization Mode
191	0	ESI

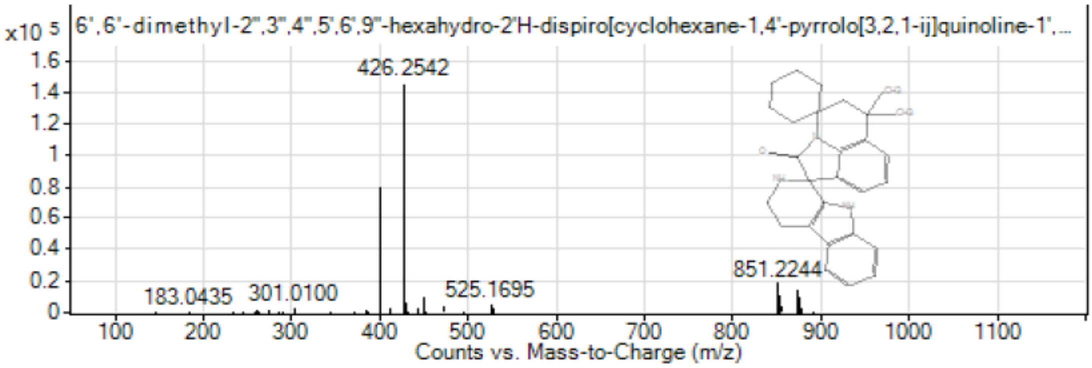

Figure S9: HPLC-HRMS-ESI spectra of 6',6'-dimethyl-2,3,4,5',6',9-hexahydrodispiro[*β*-carboline-1,1'-pyrrolo[3,2,1-*ij*]quinoline-4',1''-cyclohexan]-2'-one **41453.**

Qualitative Analysis Report

**Data Filename**	2330.d	**Sample Name**	
**Sample Type**	Sample	**Position**	Vial 79
**Instrument Name**	Instrument 1	**User Name**	
**Acq Method**	ACN-H2O_60-40.m	**Acquired Time**	12/30/2021 3:37:02 PM
**IRM Calibration Status**	Success	**DA Method**	111.m
**Comment**			

**Sample Group**		**Info.**	
**Stream Name**	LC 1	**Acquisition SW Version**	6200 series TOF/6500 series Q-TOF B.06.01 (B6172 SP1)

User Chromatograms

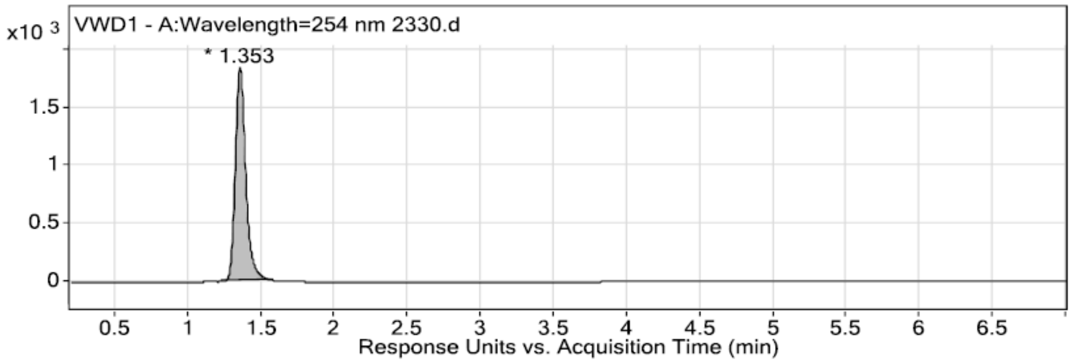

Integration Peak List

Peak	Start	RT	End	Height	Area	Area %
1	1.227	1.353	1.587	1844.29	9055.79	100

User Spectra

Fragmentor Voltage	Collision Energy	Ionization Mode
191	0	ESI

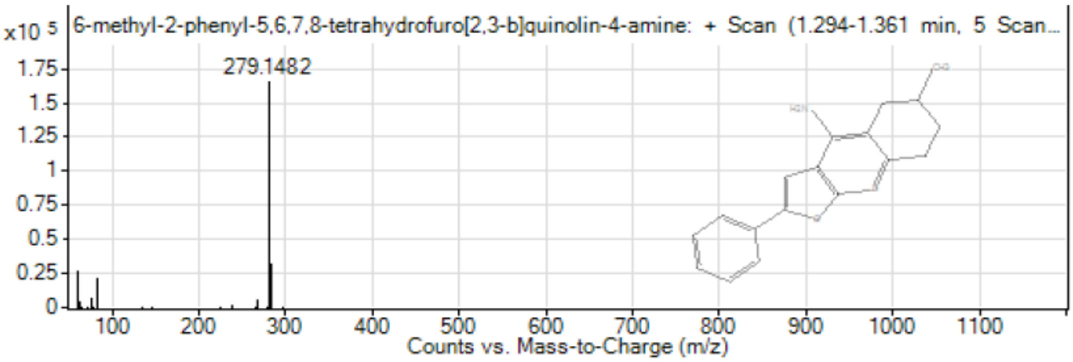

Figure S10: HPLC-HRMS-ESI spectra of 6-methyl-2-phenyl-5,6,7,8-tetrahydrofuro[2,3-*b*]quinolin-4-amine **224530.**

Qualitative Analysis Report

**Data Filename**	2326.d	**Sample Name**	
**Sample Type**	Sample	**Position**	Vial 75
**Instrument Name**	Instrument 1	**User Name**	
**Acq Method**	ACN-H2O_60-40.m	**Acquired Time**	12/30/2021 2:49:08 PM
**IRM Calibration Status**	Success	**DA Method**	111.m
**Comment**			

**Sample Group**		**Info.**	
**Stream Name**	LC 1	**Acquisition SW Version**	6200 series TOF/6500 series Q-TOF B.06.01 (B6172 SP1)

User Chromatograms

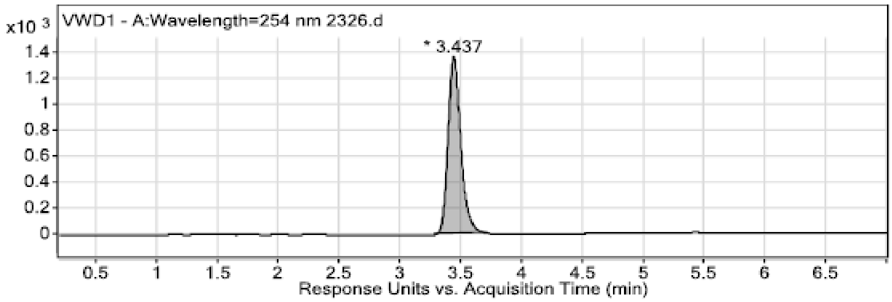

Integration Peak List

Peak	Start	RT	End	Height	Area	Area %
1	3.283	3.437	3.723	1372.38	9877.87	100

User Spectra

Fragmentor Voltage	Collision Energy	Ionization Mode
191	0	ESI

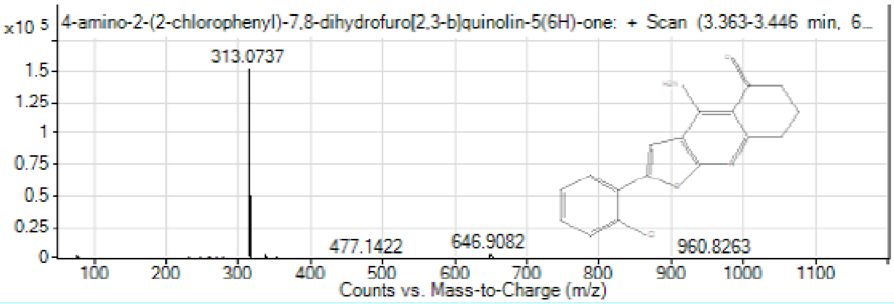

Figure S11: HPLC-HRMS-ESI spectra of 4-amino-2-(2-chlorophenyl)-7,8-dihydrofuro[2,3-*b*]quinolin-5(6H)-one **224533.**

Qualitative Analysis Report

**Data Filename**	2324.d	**Sample Name**	
**Sample Type**	Sample	**Position**	Vial 73
**Instrument Name**	Instrument 1	**User Name**	
**Acq Method**	ACN-H2O_60-40.m	**Acquired Time**	12/30/2021 1:50:43 PM
**IRM Calibration Status**	Success	**DA Method**	111.m
**Comment**			

**Sample Group**		**Info.**	
**Stream Name**	LC 1	**Acquisition SW Version**	6200 series TOF/6500 series Q-TOF B.06.01 (B6172 SP1)

User Chromatograms

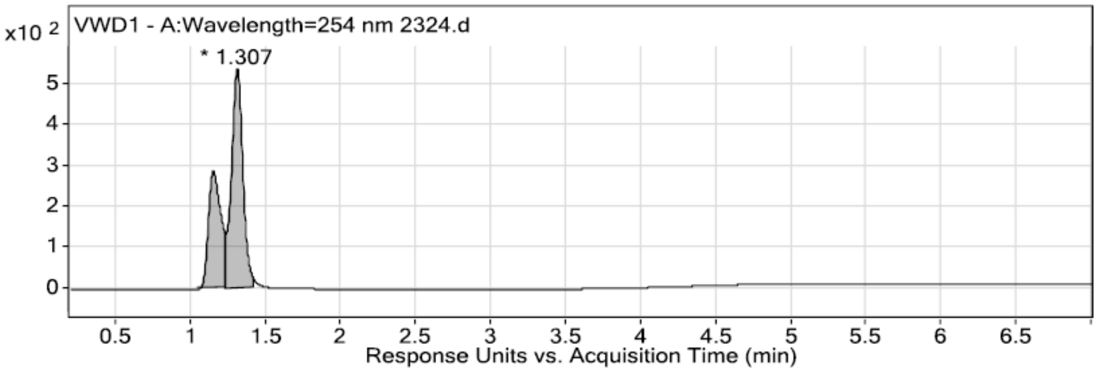

Integration Peak List

Peak	Start	RT	End	Height	Area	Area %
1	1.047	1.15	1.233	287.24	1680.27	59.36
2	1.233	1.307	1.42	538.63	2830.57	100

User Spectra

Fragmentor Voltage	Collision Energy	Ionization Mode
191	0	ESI

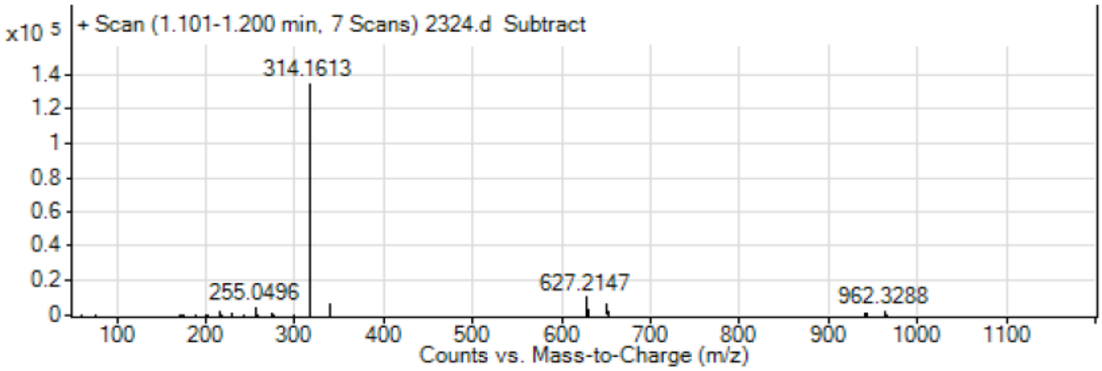

Peak List

m/z	z	Abund
213.0418	1	3343.32
255.0496	1	5682.67
314.1613	1	134947.63
315.0924	1	24918
316.0943	1	2734.27

Qualitative Analysis Report

336.0702	1	7437.8
627.2147	1	10939.52
628.2147	1	4410.44
649.1942	1	6411.3
962.3288	1	3157.59

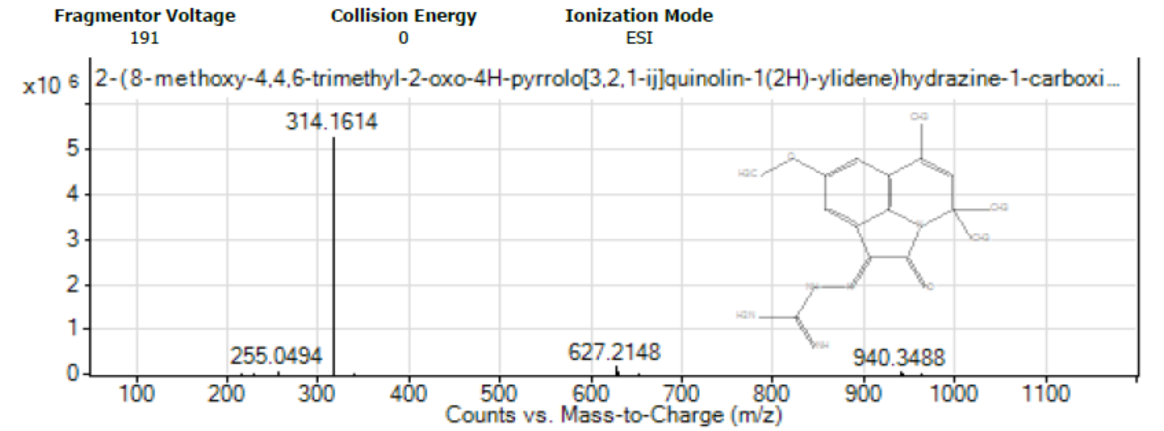

Figure S12: HPLC-HRMS-ESI spectra of 2-(8-methoxy-4,4,6-trimethyl-2-oxo-4*H*-pyrrolo[3,2,1-*ij*]quinolin-1(2*H*)-ylidene)hydrazinecarboximidamide **225635.**

Qualitative Analysis Report

**Data Filename**	2323.d	**Sample Name**	
**Sample Type**	Sample	**Position**	Vial 72
**Instrument Name**	Instrument 1	**User Name**	
**Acq Method**	ACN-H2O_60-40.m	**Acquired Time**	12/30/2021 1:38:50 PM
**IRM Calibration Status**	Success	**DA Method**	111.m
**Comment**			

**Sample Group**		**Info.**	
**Stream Name**	LC 1	**Acquisition SW Version**	6200 series TOF/6500 series Q-TOF B.06.01 (B6172 SP1)

User Chromatograms

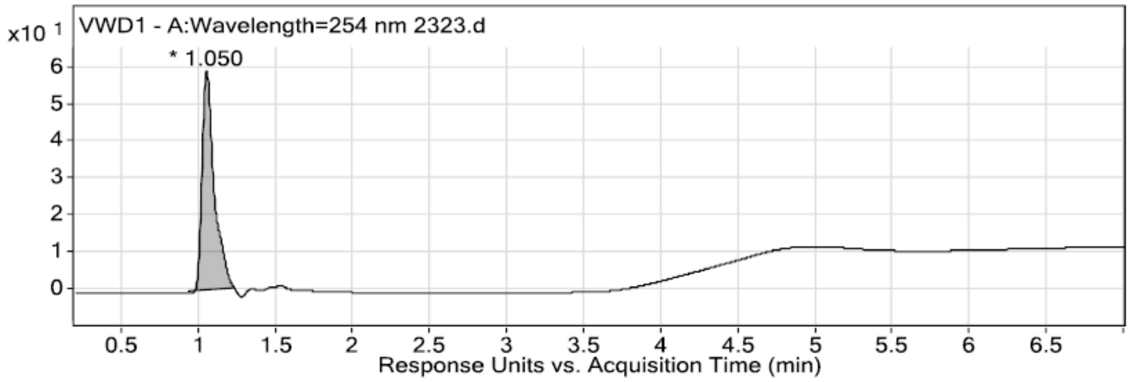

Integration Peak List

Peak	Start	RT	End	Height	Area	Area %
1	0.933	1.05	1.233	59.69	327.2	100

User Spectra

**Fragmentor Voltage**	**Collision Energy**	**Ionization Mode**
191	0	ESI

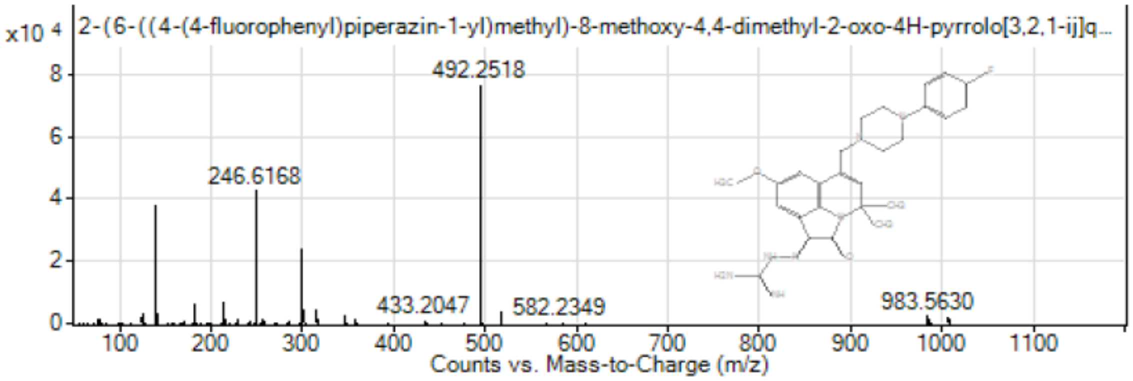

Figure S13: HPLC-HRMS-ESI spectra of 2-[6-{[4-(4-fluorophenyl)piperazin-1-yl]methyl}-8-methoxy-4,4-dimethyl-2-oxo-4*H*-pyrrolo[3,2,1-*ij*]quinolin-1(2*H*)-ylidene]hydrazinecarboximidamide **225638.**

Qualitative Analysis Report

**Data Filename**	2327.d	**Sample Name**	
**Sample Type**	Sample	**Position**	Vial 76
**Instrument Name**	Instrument 1	**User Name**	
**Acq Method**	ACN-H2O_60-40.m	**Acquired Time**	12/30/2021 3:01:05 PM
**IRM Calibration Status**	Success	**DA Method**	111.m
**Comment**			

**Sample Group**		**Info.**	
**Stream Name**	LC 1	**Acquisition SW Version**	6200 series TOF/6500 series Q-TOF B.06.01 (B6172 SP1)

User Chromatograms

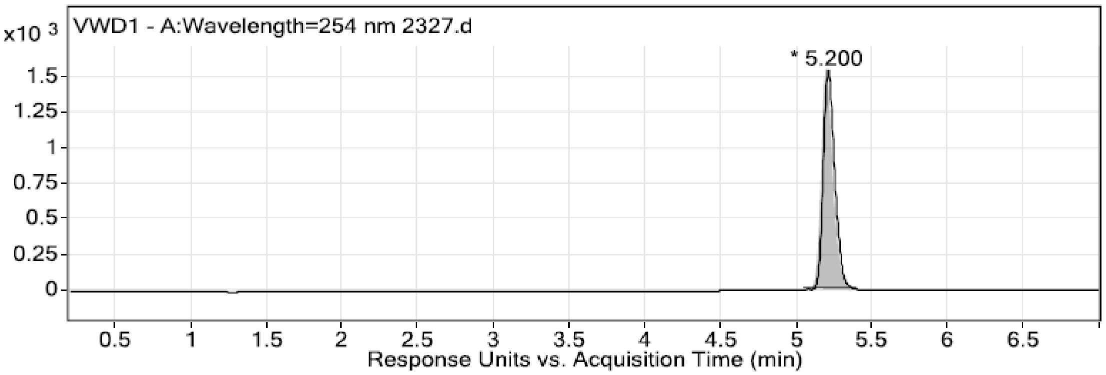

Integration Peak List

Peak	Start	RT	End	Height	Area	Area %
1	5.05	5.2	5.4	1532.92	8100.2	100

User Spectra

Fragmentor Voltage	Collision Energy	Ionization Mode
191	0	ESI

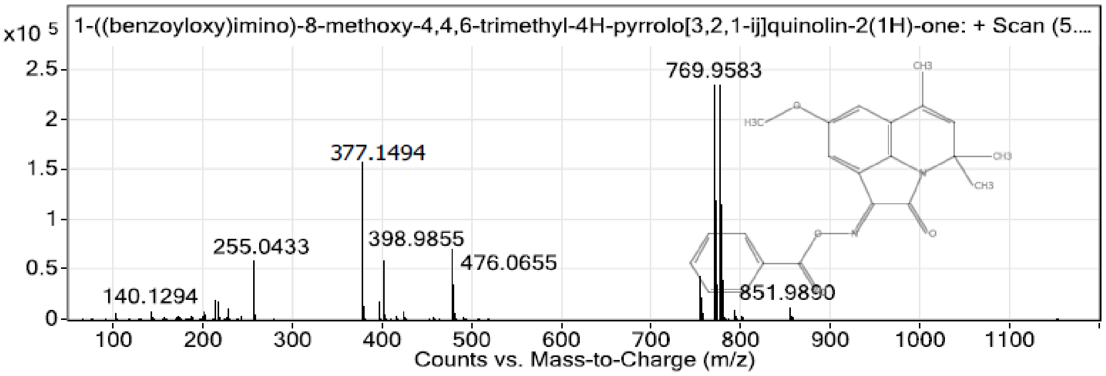

Peak List

m/z	z	Abund
255.0433	1	116605.66
377.1494	1	159291.66
398.9855	1	60576.05
477.0682	1	26812.1
752.9418	1	45277.84
769.9583	1	234643.83

Figure S14: HPLC-HRMS-ESI spectra of 8-methoxy-4,4,6-trimethyl-4*H*-pyrrolo[3,2,1-*ij*]quinoline-1,2-dione1-(*O*-benzoyloxime) **225746**.

Qualitative Compound Report

**Data File**	LCMS_3597.d	**Sample Name**	#2099
**Sample Type**	Sample	**Position**	Vial 99
**Instrument Name**	Instrument 1	**User Name**	
**Acq Method**	ACN-H2O_50-50.m	**Acquired Time**	18-APR-20 4:03:51 PM
**IRM Calibration Status**	Success	**DA Method**	13032017.m
**Comment**			

**Stream Name**	LC 1	**Acquisition SW Version**	6200 series TOF/6500 series Q-TOF B.06.01 (B6157)

Fragmentor Voltage 191 **Collision Energy** 0 **Ionization Mode** ESI

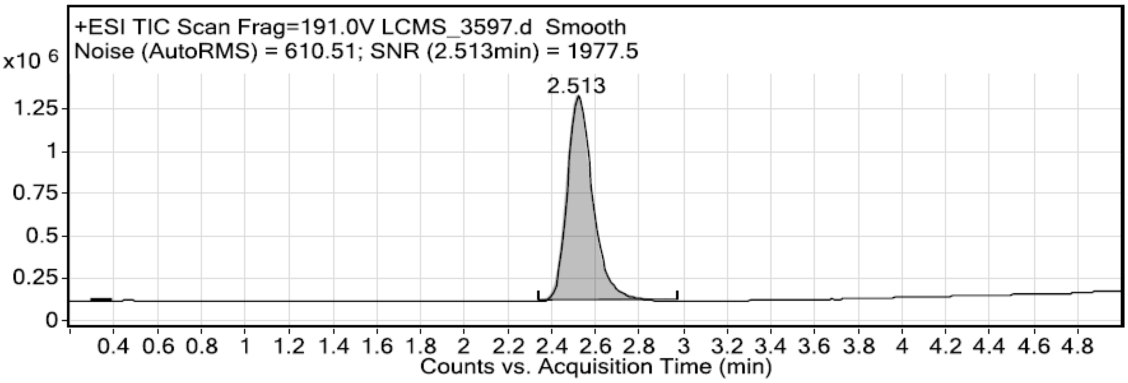

User Chromatogram Peak List

RT	Height	Height %	Area	Area %	Area Sum %	S/N	Symmetry	Width
2.513	1207309.3	100	9935730.02	100	100	1977.5	1.52	0.636

Compound Table

Compound Label	RT	Name	Formula	MFG Formula	DB Formula
Cpd 1: 6,8,8,9-tetramethyl-3-(2-morpholino-6-oxo-1,6-dihydropyrimidin-4-yl)-6,7,8,9-tetrahydro-2H-pyrano[3,2-g]quinolin-2-one	2.513	6,8,8,9-tetramethyl-3-(2-morpholino-6-oxo-1,6-dihydropyrimidin-4-yl)-6,7,8,9-tetrahydro-2H-pyrano[3,2-g]quinolin-2-one	C24H28N4O4	C24H28N4O4	C24H28N4O4

Compound Label	Name	RT	Algorithm
Cpd 1: 6,8,8,9-tetramethyl-3-(2-morpholino-6-oxo-1,6-dihydropyrimidin-4-yl)-6,7,8,9-tetrahydro-2H-pyrano[3,2-g]quinolin-2-	**6,8,8,9-tetramethyl-3-(2-morpholino-6-oxo-1,6-dihydropyrimidin-4-yl)-6,7,8,9-tetrahydro-2H-pyrano[3,2-g]quinolin-**	2.513	Spectrum Extraction

MS Spectrum

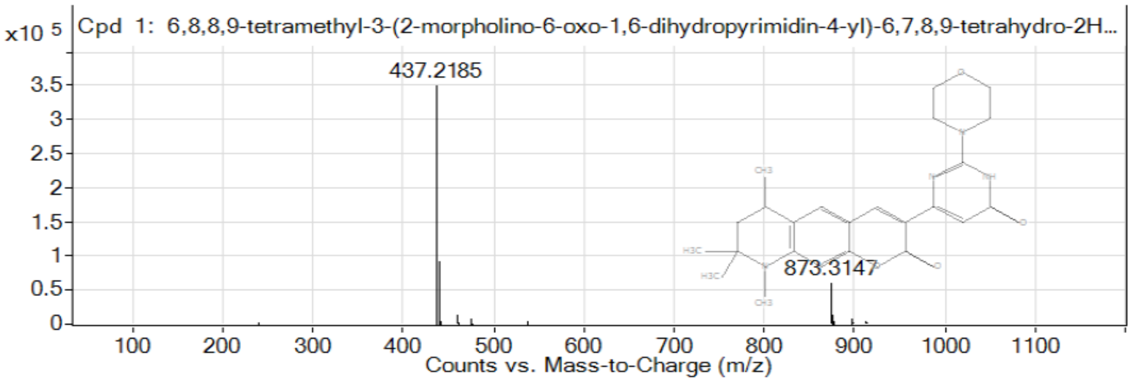

Figure S15: HPLC-HRMS-ESI spectra of 6,8,8,9-tetramethyl-3-(2-morpholin-4-yl-6-oxo-1,6-dihydropyrimidin-4-yl)-6,7,8,9-tetrahydro-2*H*-pyrano[3,2-*g*]quinolin-2-one **225006**

Qualitative Compound Report

**Data File**	LCMS_6043.d	**Sample Name**	
**Sample Type**	Sample	**Position**	Vial 20
**Instrument Name**	Instrument 1	**User Name**	
**Acq Method**	ACN-H2O_60-40.m	**Acquired Time**	16-Jan-20 1:40:57 PM
**IRM Calibration Status**	Success	**DA Method**	13032017.m
**Comment**			

**Sample Group**		**Info.**	
**Stream Name**	LC 1	**Acquisition SW Version**	6200 series TOF/6500 series Q-TOF B.06.01 (B6157)

Fragmentor Voltage 191 **Collision Energy** 0 **Ionization Mode** ESI

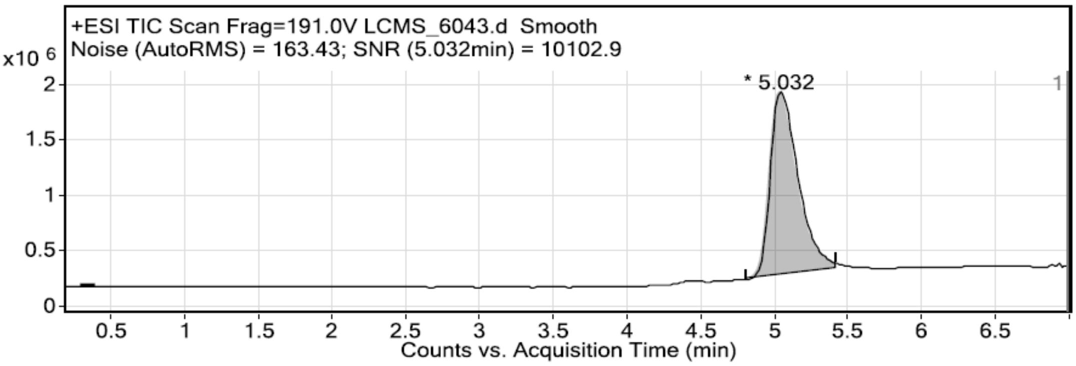

User Chromatogram Peak List

RT	Height	Height %	Area	Area %	Area Sum %	S/N	Symmetry	Width
5.032	1651095.49	100	22050065.21	100	100	10102.9	2.36	0.613

Compound Table

Compound Label	RT	Name	Formula	MFG Formula	DB Formula
Cpd 1: 3-(4-methoxybenzoyl)-6,8,8,9-tetramethyl-6,7,8,9-tetrahydro-2H-pyrano[3,2-g]quinolin-2-one	5.032	3-(4-methoxybenzoyl)-6,8,8,9-tetramethyl-6,7,8,9-tetrahydro-2H-pyrano[3,2-g]quinolin-2-one	C24H25NO4	C24H25NO4	C24H25NO4

Compound Label	Name	RT	Algorithm
Cpd 1: 3-(4-methoxybenzoyl)-6,8,8,9-tetramethyl-6,7,8,9-tetrahydro-2H-pyrano[3,2-g]quinolin-2-one	**3-(4-methoxybenzoyl)-6,8,8,9-tetramethyl-6,7,8,9-tetrahydro-2H-pyrano[3,2-g]quinolin-2-one**	5.032	Spectrum Extraction

MS Spectrum

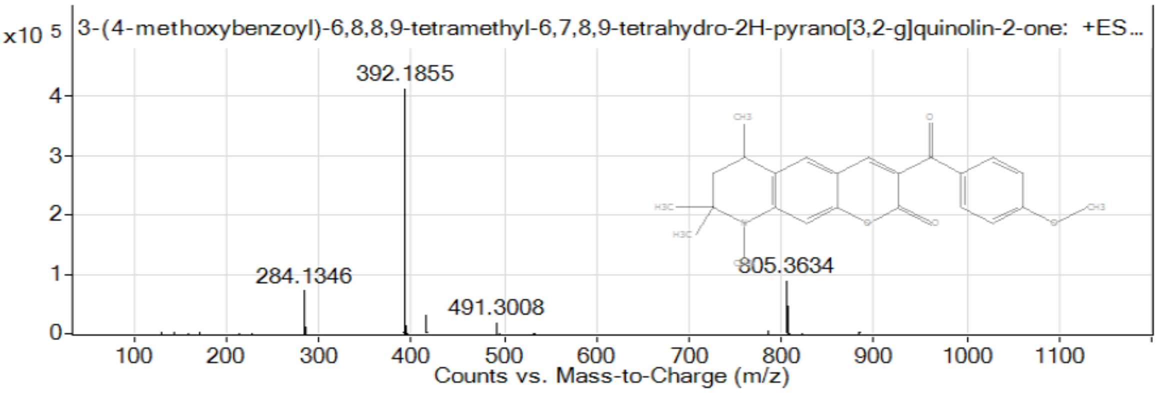

Figure S16: HPLC-HRMS-ESI spectra of 3-(4-methoxybenzoyl)-6,8,8,9-tetramethyl-6,7,8,9-tetrahydro-2*H*-pyrano[3,2-*g*]quinolin-2-one **224870.**

Qualitative Compound Report

**Data File**	LCMS_6558.d	**Sample Name**	
**Sample Type**	Sample	**Position**	Vial 16
**Instrument Name**	Instrument 1	**User Name**	
**Acq Method**	ACN-H2O_60-40.m	**Acquired Time**	25-Mar-20 2:08:33 PM
**IRM Calibration Status**	Success	**DA Method**	13032017.m
**Comment**			

**Sample Group**		**Info.**	
**Stream Name**	LC 1	**Acquisition SW Version**	6200 series TOF/6500 series Q-TOF B.06.01 (B6157)

Fragmentor Voltage 191 **Collision Energy** 0 **Ionization Mode** ESI

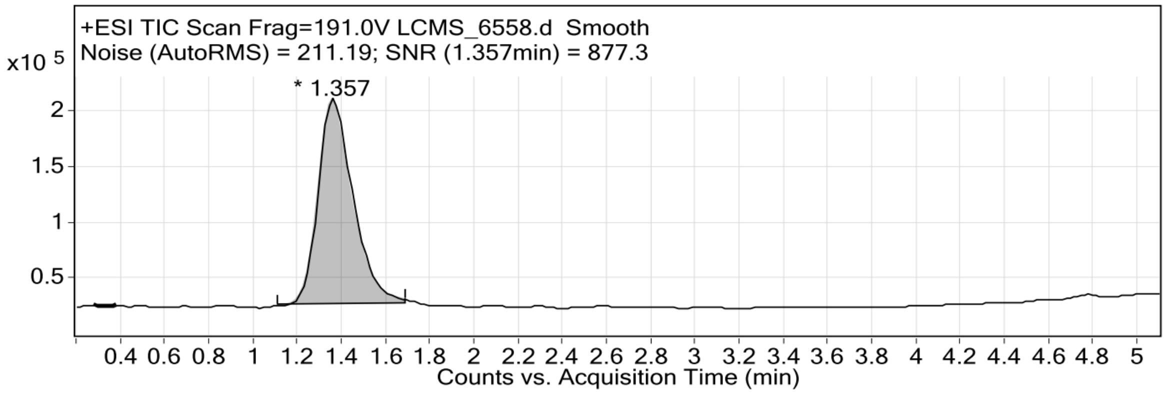

User Chromatogram Peak List

RT	Height	Height %	Area	Area %	Area Sum %	S/N	Symmetry	Width
1.357	185269.19	100	2056118.26	100	100	877.3	1.58	0.58

Compound Table

Compound Label	RT	Name	Formula	MFG Formula	DB Formula
Cpd 1: 6-((4-(benzo[d][1,3]dioxol-5-ylmethyl)piperazin-1-yl)methyl)-8-fluoro-4,4-dimethyl-1H-pyrrolo[3,2,1-ij]quinoline-1,2(4H)-dione	1.357	6-((4-(benzo[d][1,3]dioxol-5-ylmethyl)piperazin-1-yl)methyl)-8-fluoro-4,4-dimethyl-1H-pyrrolo[3,2,1-ij]quinoline-1,2(4H)-dione	C26H26FN3O4	C26H26FN3O4	C26H26FN3O4

Compound Label	Name	RT	Algorithm
Cpd 1: 6-((4-(benzo[d][1,3]dioxol-5-ylmethyl)piperazin-1-yl)methyl)-8-fluoro-4,4-dimethyl-1H-pyrrolo[3,2,1-ij]quinoline-1,2(4H)-dione	**6-((4-(benzo[d][1,3]dioxol-5-ylmethyl)piperazin-1-yl)methyl)-8-fluoro-4,4-dimethyl-1H-pyrrolo[3,2,1-**	1.357	Spectrum Extraction

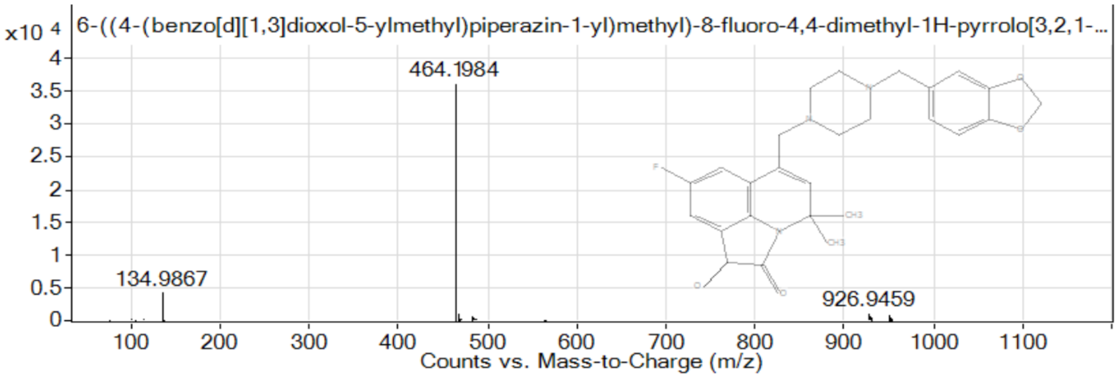

Figure S17: HPLC-HRMS-ESI spectra of 6-((4-benzo[*d*][1,3]dioxol-5-ylmethyl)piperazine-1-yl)methyl)-8-fluoro-4,4-dimethyl–1*H*-pyrrolo[3,2,1-*ij*]quinolin-1,2(4*H*)-dione **225624.**

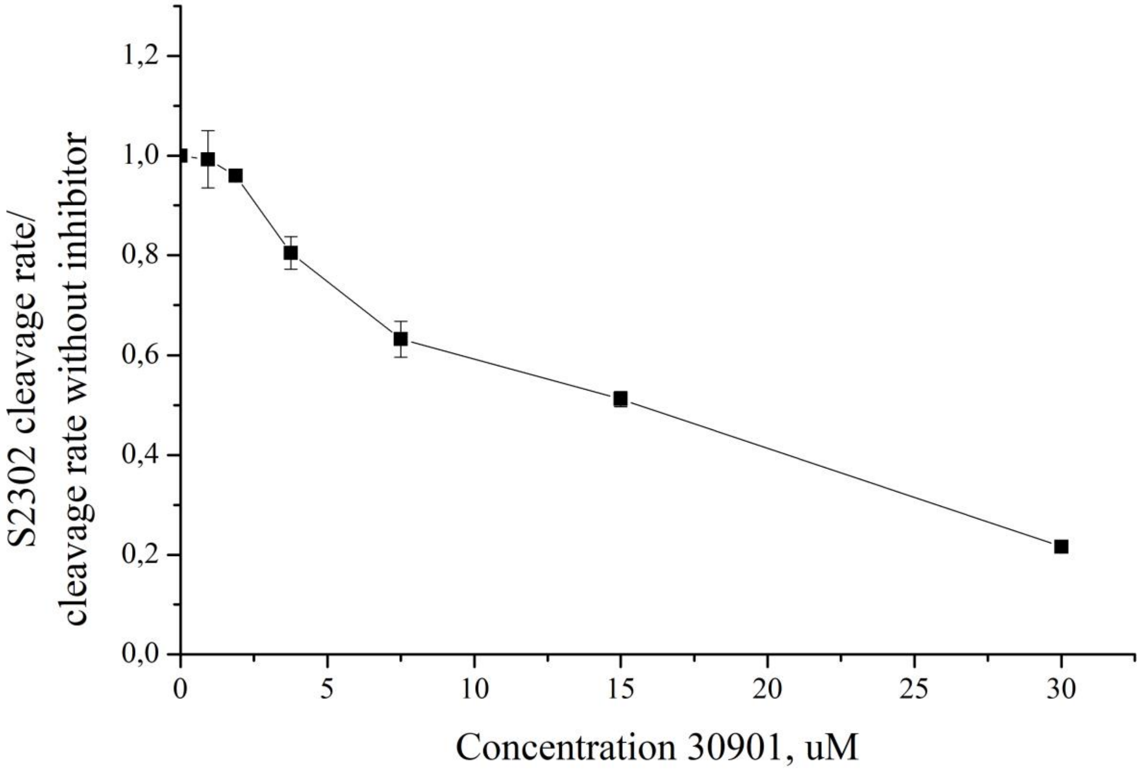

Figure S18. Inhibition curve for **30901** against factor XIIa. The dots are mean ± SEM.

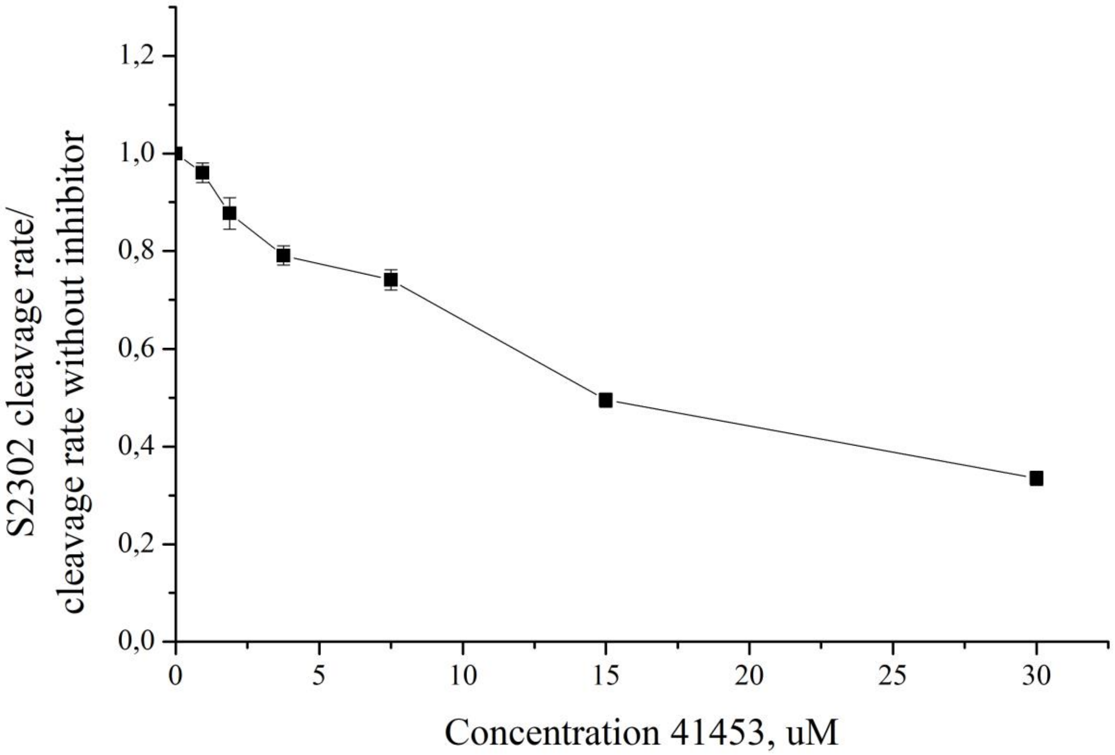

Figure S19. Inhibition curve for **41453** against factor XIIa. The dots are mean ± SEM.

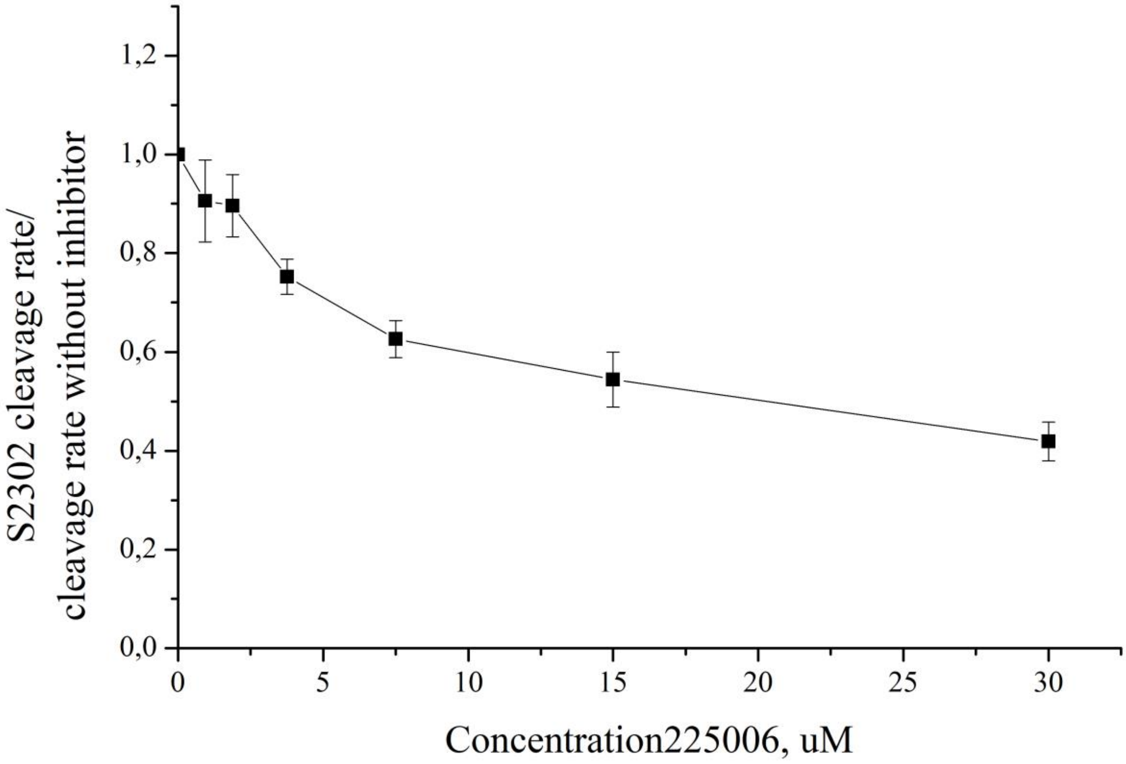

Figure S20. Inhibition curve for **225006** against factor XIIa. The dots are mean ± SEM.

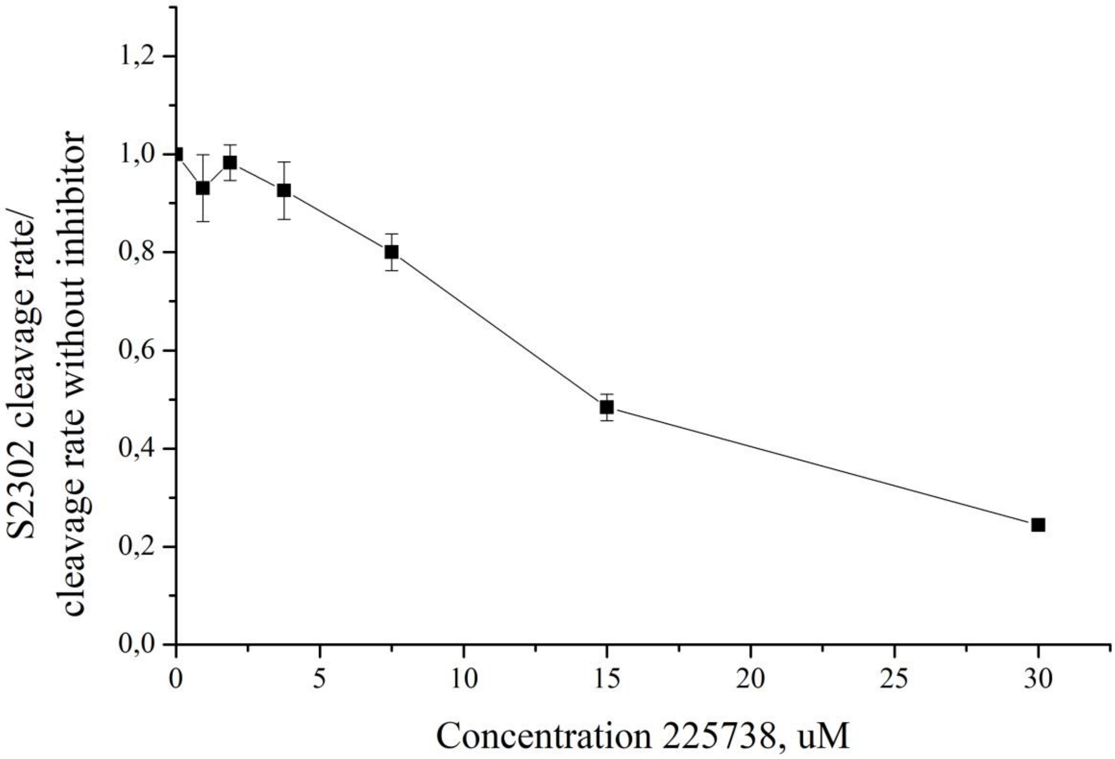

Figure S21. Inhibition curve for **225738** against factor XIIa. The dots are mean ± SEM.

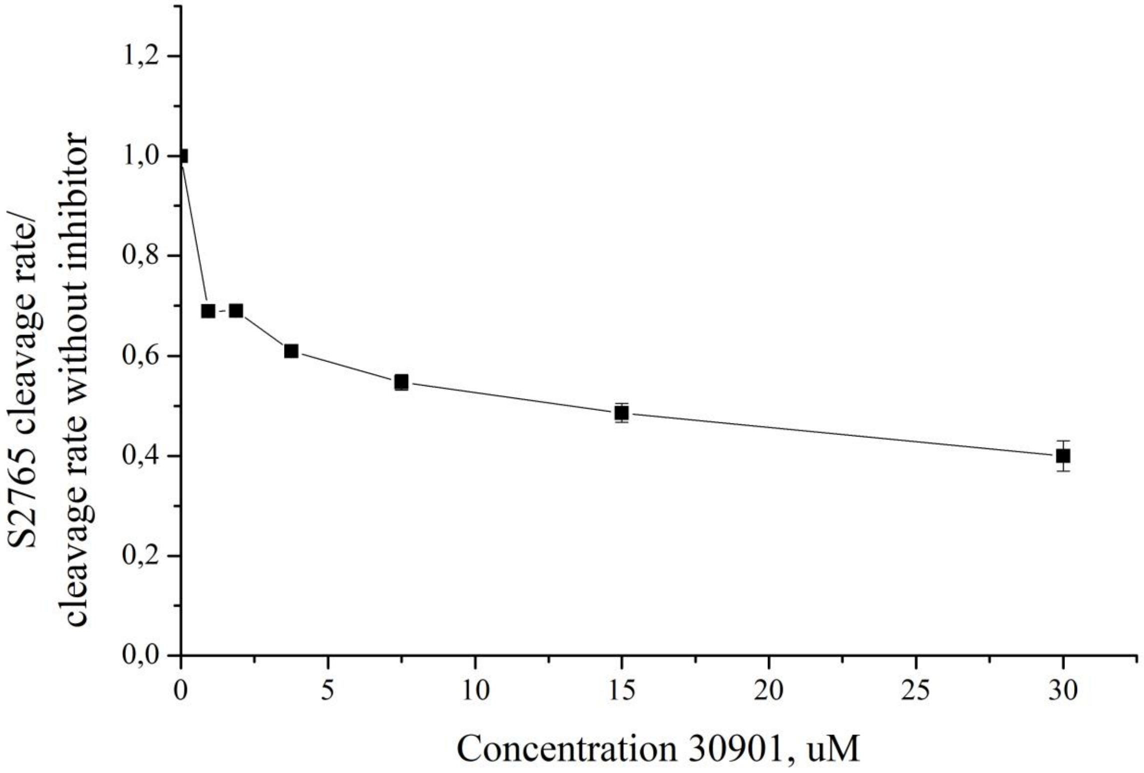

Figure S22. Inhibition curve for **30901** against factor Xa. The dots are mean ± SEM.
